# Supplementary material for: GWAS meta-analysis of over 29,000 people with epilepsy identifies 26 risk loci and subtype-specific genetic architecture
Source: Nat Genet. 2023 Aug 31;55(9):1471–82. doi: 10.1038/s41588-023-01485-w (PMC10484785; doi:10.1038/s41588-023-01485-w)
Supplement: Supplementary file 1 — Supplementary Tables 1–16, Supplementary Figs. 1–26, Supplementary Note, source code for Fig. 2 and Supplementary References. [file 41588_2023_1485_MOESM1_ESM.pdf]

nature genetics

Article

https://doi.org/10.1038/s41588-023-01485-w

GWAS meta-analysis of over 29,000 people with epilepsy identifies 26 risk loci and subtype-specific genetic architecture

In the format provided by the authors and unedited

Table of contents

Supplementary Tables 2
- Supplementary table 1. 3
- Supplementary table 2. 3
- Supplementary table 3. 4
- Supplementary table 4. 5
- Supplementary table 5. 6
- Supplementary table 6. 6
- Supplementary table 7. 7
- Supplementary table 8. 7
- Supplementary table 9. 8
- Supplementary table 10. 8
- Supplementary table 11. 8
- Supplementary table 12. 9
- Supplementary table 13. 10
- Supplementary table 14. 11
- Supplementary table 15. 11
- Supplementary table 16. 11

Supplementary Figures 12
- Supplementary figure 1. 12
- Supplementary figure 2. 13
- Supplementary figure 3. 14
- Supplementary figure 4. 15
- Supplementary figure 5. 16
- Supplementary figure 6. 17
- Supplementary figure 7. 17
- Supplementary figure 8. 18
- Supplementary figure 9. 19
- Supplementary figure 10. 20
- Supplementary figure 11. 20
- Supplementary figure 12. 21
- Supplementary figure 13. 22
- Supplementary figure 14. 22
- Supplementary figure 15. 23
- Supplementary figure 16. 24
- Supplementary figure 17. 25
- Supplementary figure 18. 26
- Supplementary figure 19. 27
- Supplementary figure 20. 28
- Supplementary figure 21. 29
- Supplementary figure 22. 30
- Supplementary figure 23. 31
- Supplementary figure 24. 32
- Supplementary figure 25. 33
- Supplementary figure 26. 34

Supplementary Note 35

Source code for Figure 2 37

Supplementary References 37

Supplementary Tables

Cohort	Genotyping platform	Ethics Committee / IRB	Ancestry	Phenotype	Cases
**Epi25^**	**Illumina Infinium GSA**	Massachusetts General Brigham (formerly Partners) Institutional Review Board (2012P000788, 2014P000938, 2014P001342, 2013P002634)	European	All epilepsy	11544
				GGE	3153
				Focal	4523
				Unclassified	3868
				Control	13121
			European: Finnish	All epilepsy	474
				GGE	91
				Focal	314
				Unclassified	-
				Control	986
			African	All epilepsy	612
				GGE	276
				Focal	230
				Unclassified	106
				Control	3838
			Asian: HK	All epilepsy	839
				GGE	55
				Focal	639
				Unclassified	145
				Control	594
			Asian: JPN	All epilepsy	256
				GGE	63
				Focal	4
				Unclassified	189
				Control	211
**Norwegian GenEpA**	**Illumina Human610-Quadv1**	Regional committees for medical and health research ethics, Norway (REK) (reference number: S-01271)	European	All epilepsy	201
				Focal	201
				Control	-
**Swiss GenEpA**	**Illumina Human610-Quadv1**	Ethics committee of the Canton of Zurich ("Kantonale Ethikkommission, Kanton Zürich")	European	All epilepsy	231
				Focal	231
				Control	259
**Janssen Pharmaceuticals**	**Illumina 1M**	NA*	European	All epilepsy	410
				Focal	410
				Control	3016
**Austrian GenEpA**	**Illumina Human CNV 370 duo**	Ethics Committee of the Medical University of Vienna	European	All epilepsy	165
				Focal	165
				Control	337

Supplementary table 1.
Overview of novel cohorts, genotyping array, relevant ethics/IRB approvals, ancestry, and sample sizes. HK: Hong Kong; JPN: Japan. These details for previously published cohorts included in this current manuscript can be found in the supplements of our 2018 study.[1] ^ Please see Supplementary Information below for detailed descriptions of control cohorts used for Epi25 analyses. * The Janssen clinical studies were carried out in accordance with the ethical principles outlined in the Declaration of Helsinki, Good Clinical Practices guidelines, and applicable regulatory requirements. The study protocols were approved by the local, regional, or central Institutional Review Board (IRB) or Independent Ethics Committee (IEC) overseeing the numerous clinical sites involved in multi-centre pharmaceutical trials.

**Contributor**	**Ancestry**	**Description**	**n**	**Platform Chip**
BRAINN/UNICAMP	Brasilian (Afr&Eur)	Both	340	Affymetrix 6.0
CHoP	US (Afr&Eur)	Both	11071	Illumina 550, Omni-Express
Duke University	US (Eur)	Case	777	Illumina 610
EPGP	US (Eur)	Case	1298	Illumina HumanCore
EPICURE	NW European	Case	2282	Affymetrix 6.0
EpiPGX	NW European	Case	5031	Illumina Omni-Express
GenEpa	Finnish	Both	715	Illumina 610
German controls	German	Control	1317	Illumina OmniExpress
Helsinki Birth Cohort	Finnish	Control	1586	Illumina 610
KORA	NW European	Control	1331	Affymetrix 6.0
Imperial-Liverpool-Melbourne	British	Case	1294	Illumina Omni-Express
Northwest-Europeans	NW European	Control	1622	Illumina XX
POBI	British	Control	2706	Illumina 1.2M
PopGen	NW European	Control	1062	Affymetrix 6.0
RCSI	Irish	Case	645	Illumina 610
Royal Melbourne Hospital	Australian (Eur)	Case	348	Illumina 610, Omni-Express
Trinity Student Study	Irish	Control	2232	Illumina Omni1-Quad
UCL	British	Case	1051	Illumina 610
UK National Blood Service	British	Control	2501	Illumina 1.2M
ULB	Belgian	Case	539	Illumina 610
University of Bonn	German	Case	284	Illumina XX
University of Hong Kong	Han Chinese	Both	3363	Illumina 550, 610
Wellcome 1958 Birth Cohort	British	Control	2699	Illumina 1.2M

Supplementary table 2.
Details of participating cohorts from our previously published GWAS.[1] Numbers of cases and controls are after quality control filtering.

Phenotype	Sub-phenotype description	n	EUR	ASI	AFR
GGE	Generalized Epilepsy, not otherwise specified, with spike and wave EEG	3352	3024	44	284
	Childhood Absence Epilepsy (CAE)	1072	1049	6	17
	Juvenile Absence Epilepsy (JAE)	671	662	4	5
	Juvenile Myoclonic Epilepsy (JME)	1813	1732	61	20
	GTCS only, with spike and wave EEG	499	485	3	11
	**Subtotal**	**7407**	**6952**	**118**	**337**
Focal	Focal Epilepsy, not otherwise specified	3981	3688	140	153
	Focal Epilepsy, documented lesion negative	6367	5778	466	123
	Focal Epilepsy, documented hippocampal sclerosis (HS)	1375	1260	107	8
	Focal Epilepsy, documented lesion other than HS	4661	4213	416	32
	**Subtotal**	**16384**	**14939**	**1129**	**316**
Unclassified	Epilepsy, not otherwise specified	6153	5668	379	106
	**Cases**	**29944**	**27559**	**1626**	**759**
	**Controls**	**52538**	**42436**	**3680**	**6422**
	**Total subjects**	**82482**	**69995**	**5306**	**7181**

Supplementary table 3.
Overview of number of cases and controls, stratified by phenotype and ancestry.

Phenotype	Locus name	Locus position (hg19)	Lead SNP	Number of independent significant SNPs	Independent significant SNPs
All epilepsy	2p16.1	chr2:57917222-58505679	rs13032423	1	rs13032423
	2q24.3	chr2:166716305-167124221	rs59237858	2	rs59237858; rs1960242
	9q21.13	chr9:76297313-76625089	rs4744696	1	rs4744696
	10q24.32	chr10:103493226-103989812	rs3740422	1	rs3740422
GGE	1q43	chr1:237846053-237908911	rs876793	1	rs876793
	2p16.1	chr2:57917222-58756729	rs11688767	3	rs11688767; rs77876353; rs13416557
	2q12.1	chr2:104056769-104481325	rs62151809	1	rs62151809
	2q24.3	chr2:166818404-166994996	rs11890028	1	rs11890028
	2q32.2	chr2:191504467-191710069	rs6721964	1	rs6721964
	3p22.3	chr3:36218075-36345769	rs9861238	1	rs9861238
	3p21.31	chr3:50184538-50421081	rs739431	1	rs739431
	4p15.1	chr4:31107765-31204950	rs1463849	1	rs1463849
	5q22.3	chr5:113837198-114440966	rs4596374	1	rs4596374
	5q31.2	chr5:136459562-136684519	rs2905552	1	rs2905552
	6q22.33	chr6:128302874-128333682	rs13219424	1	rs13219424
	7p14.1	chr7:41334517-41411165	rs37276	1	rs37276
	9q21.32	chr9:86320233-86694759	rs2780103	1	rs2780103
	10q24.32	chr10:103493226-103989812	rs11191156	1	rs11191156
	12q13.13	chr12:52319584-52348259	rs114131287	2	rs4762030; rs10431492
	16p13.3	chr16:7285674-7442293	rs62014006	1	rs62014006
	17p13.1	chr17:8036060-8219478	rs2585398	1	rs2585398
	17q21.32	chr17:45938105-46554456	rs16955463	1	rs16955463
	19p13.3	chr19:2102543-2136680	rs75483641	1	rs75483641
	21q21.1	chr21:21655062-21719113	rs1487946	1	rs1487946
	21q22.1	chr21:32036541-32203274	rs7277479	1	rs7277479
	22q13.32	chr22:48615721-48639993	rs469999	1	rs469999
CAE	2p16.1	chr2:57942325-58484172	rs12185644	1	rs12185644
JME	4p12	chr4:46250605-46397617	rs17537141	1	rs17537141
	8q23.1	chr8:109733213-109922163	rs3019359	1	rs3019359
	16p11.2	chr16:30603521-31275374	rs1046276	1	rs1046276

Supplementary table 4.

Summary of all genome-wide significant loci including genomic position and independent significant SNPs. We defined the locus position as the region encompassing all SNPs with two-tailed P<10-4 that were in LD (R^2>0.2) with the lead SNP.

SNP (Risk allele)	Chr.	Locus	P-value (GGE)	P-value (FE)	OR (ASSET)	P-value (ASSET)	Associated phenotype in ASSET
*rs60055328(C)	2	2q24.3	1.04e-7	9.62e-7	1.07	2.8e-10	GGE, FE
*rs4744696(G)	9	9q21.13	3.07e-7	8.63e-5	0.93	4.4e-8	GGE, FE
rs13032423(G)	2	2p16.1	2.88e-17	2.93e-3	0.85	8.9e-17	GGE only
rs3740422(G)	10	10q24.32	1.02e-13	4.07e-3	1.15	2.48e-12	GGE only

Supplementary table 5.
*Results from ASSET pleiotropy analyses for the 4 all epilepsy loci. The associated phenotype/s in ASSET reflect the phenotypes driving the ASSET signal, which could be both GGE individually. *Evidence for pleiotropy between GGE and Focal epilepsy (two-tailed P<5×10-8)*.

Annotation	Share	SD	Expected	Enrichment	SD	Z-score	P-value
Coding_UCSC	0.07	0.03	0.02	4.35	1.72	1.94	0.05
Conserved_LindbladToh	0.13	0.04	0.03	4.64	1.48	2.47	0.01
CTCF_Hoffman	0.02	0.04	0.02	0.73	1.72	0.16	0.87
DGF_ENCODE	0.17	0.10	0.14	1.20	0.69	0.29	0.77
DHS_Trynka	0.14	0.10	0.17	0.83	0.60	0.29	0.77
Enhancer_Andersson	0.00	0.02	0.00	-0.10	4.20	0.26	0.79
Enhancer_Hoffman	0.07	0.04	0.04	1.53	0.97	0.55	0.59
FetalDHS_Trynka	0.12	0.08	0.09	1.46	0.90	0.51	0.61
H3K27ac_Hnisz	0.47	0.03	0.39	1.19	0.08	2.40	0.02
H3K27ac_PGC2	0.35	0.06	0.27	1.28	0.21	1.38	0.17
H3K4me1_Trynka	0.58	0.07	0.43	1.35	0.16	2.23	0.03
H3K4me3_Trynka	0.23	0.05	0.14	1.67	0.38	1.76	0.08
H3K9ac_Trynka	0.25	0.05	0.13	1.97	0.41	2.33	0.02
Intron_UCSC	0.46	0.03	0.39	1.16	0.07	2.37	0.02
PromoterFlanking_Hoffman	0.02	0.03	0.01	2.49	3.07	0.49	0.63
Promoter_UCSC	0.08	0.04	0.05	1.75	0.75	1.00	0.32
Repressed_Hoffman	0.41	0.07	0.45	0.91	0.14	0.59	0.55
SuperEnhancer_Hnisz	0.24	0.02	0.17	1.43	0.11	3.80	0.00
TFBS_ENCODE	0.23	0.08	0.13	1.69	0.59	1.18	0.24
Transcr_Hoffman	0.42	0.06	0.35	1.19	0.16	1.17	0.24
TSS_Hoffman	0.05	0.03	0.02	2.48	1.60	0.93	0.35
UTR_3_UCSC	0.01	0.02	0.01	1.12	1.43	0.08	0.93
UTR_5_UCSC	0.02	0.02	0.01	3.53	2.74	0.92	0.36
WeakEnhancer_Hoffman	-0.04	0.04	0.02	-1.64	1.85	1.43	0.15
Super_Enhancer_Vahedi	0.03	0.01	0.02	1.35	0.38	0.93	0.35
Typical_Enhancer_Vahedi	0.03	0.01	0.02	1.22	0.53	0.41	0.68

Supplementary table 6.
Heritability enrichment of 26 functional categories, as assessed with LDAK heritability enrichment analyses. Statistical significance after correction for multiple comparisons (in bold) is defined as two-tailed P<0.05/26=0.0019.

Phenotype	Average sample size	SNPs used	Intercept	Average GWAS	Average MTAG
GGE	23230	4103957	1.036	1.272	1.270
CAE	4050	4103957	1.063	1.053	1.233
JAE	2587	4103957	1.03	1.045	1.221
JME	6564	4103957	1.04	1.115	1.199
GTCS	1930	4103957	1.018	1.022	1.228

Supplementary table 7.
LDSC intercepts for GGE and its subphenotypes. Intercepts were used as weights for estimating MTAG effect sizes

Phenotype	Locus	Lead SNP	P-value 2018 GWAS[1]	P-value current GWAS
**All epilepsy**	16q12.1	rs4638568	4,00E-08	5,06E-07
**Focal epilepsy**	2q24.3#	rs2212656	7,30E-09	4,11E-05
**GGE**	2p24.1	rs4665630	4,30E-08	4,28E-05
	4p12*	rs11943905	3,90E-08	4,80E-06
	6p22.3	rs68082256	1,70E-09	5,35E-08
**CAE**	2q22.3	rs13020210	2,40E-08	1,76E-07
**Focal epilepsy with hippocampal sclerosis**	3q25.31	rs1991545	1,30E-11	8,59E-08
	6q22.31	rs1318322	6,70E-09	2,49E-05

Supplementary table 8.
*List of significant loci from our previous GWAS[1] that were not replicated at genome-wide significance level (two-tailed P<5e-8). *In the current GWAS, this locus is genome-wide significant in the JME GWAS (p=4.6E-8). #In the current GWAS, this locus is genome-wide significant in the 'all epilepsy' (p=5.7E-12) and the GGE GWAS (1.7E-8).*

Phenotype	λ	$λ_{1000}$	Mean χ2	LDSC intercept	Attenuation ratio
All epilepsy - ILAE3	1.25	1.01	1.27	1.10	0.37
Focal epilepsy - ILAE3	1.17	1.01	1.17	1.10	0.58
GGE - ILAE3	1.26	1.02	1.35	1.04	0.11
All epilepsy - ILAE2	1.25	1.01	1.18	1.15	0.83
Focal epilepsy - ILAE2	1.20	1.02	1.28	1.18	0.64
GGE - ILAE2	1.25	1.04	1.30	1.10	0.33
All epilepsy - Epi25	1.06	1.00	1.07	1.02	0.28
Focal epilepsy- Epi25	1.04	1.01	1.04	1.01	0.15
GGE - Epi25	1.05	1.01	1.06	0.97	<0

Supplementary table 9.
Estimation of inflation factor and the LD-score regression intercept stratified by phenotype. We compared the European only analyses from the current GWAS ('ILAE3') with our previously published GWAS ('ILAE2') and the Epi25 cohort (which constitutes the far majority of additional cases). λ: genomic inflation factor, $λ_{1000}$: genomic inflation factor corrected for an equivalent study of 1000 cases and 1000 controls. Attenuation ratio is calculated as (LDSC intercept – 1)/(Mean χ2 – 1).

Phenotype	cases	controls	K: prevalence	Z	Observed-scale heritability	Liability scale heritability
All epilepsy	27559	42436	0.005	0.0145	0.3733	0.177 (0.155 - 0.199)
Focal epilepsy	14939	42436	0.003	0.0091	0.3733	0.160 (0.140 - 0.180)
GGE	6952	42436	0.002	0.0063	0.9955	0.395 (0.343 - 0.446)
JME	1728	37339	0.00035	0.0013	2.1135	0.635 (0.510 - 0.760)
JAE	662	37339	0.00015	0.0006	3.3528	0.900 (0.633 - 1.166)
CAE	1049	37339	0.00015	0.0006	3.0427	0.816 (0.638 - 0.995)
GTCSA	485	37339	0.0002	0.0008	1.7824	0.496 (0.140 - 0.853)
Focal HS	1260	37339	0.00075	0.0026	1.4020	0.472 (0.294 - 0.649)
Focal other lesion	4213	37339	0.00135	0.0044	0.6778	0.251 (0.188 - 0.313)
Focal non-lesional	5778	37339	0.0009	0.0031	0.2452	0.085 (0.046 -0.124)

Supplementary table 10.
SNP-based heritabilities as calculated by LDAK, with the BLD-LDAK model. Observed-scale heritability is calculated using effective-sample sizes, after which it was converted to liability-scale heritability using the same prevalence estimates as our previous GWAS.[1]

	GGE	CAE	JAE	JME	GTCSA
**GGE**	1	0.932	0.914	0.833	0.932
**CAE**		1	0.932	0.8	0.932
**JAE**			1	0.644	0.856
**JME**				1	0.715
**GTCSA**					1

Supplementary table 11.
LDSC genetic correlation estimates for GGE subphenotypes

Broad trait	Trait	Publication	Notes
Psychiatric	Bipolar disorder	Mullins *et al* 2021[2]	
Psychiatric	ADHD	Demontis *et al* 2019[3]	
Psychiatric	ASD	Grove *et al* 2019[4]	
Psychiatric	Schizophrenia	Trubetskoy *et al* 2022[5]	
Psychiatric	Depression	Howard *et al* 2019[6]	exc. UKBB and 23andMe
Neurological	Febrile seizures	Skotte *et al* 2022[7]	
Neurological	Parkinson's disease	Nalls *et al* 2019[8]	exc. 23andMe
Neurological	Alzheimer's disease	Wightman *et al* 2021[9]	exc. 23andMe
Neurological	Stroke	Malik *et al* 2018[10]	
Neurological	Headache	Meng *et al* 2018[11]	
Neurological / Autoimmune	Multiple sclerosis	International Multiple Sclerosis Genetics Consortium 2019[12]	
Autoimmune	Type 1 diabetes	Chiou *et al* 2021[13]	
Autoimmune	Systemic lupus erythematosus	Morris *et al* 2016[14]	
Cognitive	Intelligence	Savage, Jansen *et al* 2018[15]	
Sleep	Insomnia	Jansen *et al* 2019[16]	exc. 23andMe
Smoking	Ever smoked	Karlsson Linnér *et al* 2019[17]	
Metabolic	Type 2 diabetes	Mahajan *et al* 2018[18]	
Metabolic	Coronary disease	van der Harst, Verweij *et al* 2018[19]	

Supplementary table 12.
Phenotypes and associated publications assessed for genetic correlations with epilepsy using LDSC.

Drug	Current indication	Studies' PubMed IDs	Models
Aspirin	Pain; pyrexia; antiplatelet	11883156, 14671677, 16844276, 22765917, 28060522	Pilo, PTZ, Electro
Biperiden	Parkinson's disease	738231, 2858579	Electro, other
Captopril	Hypertension; chronic heart failure; diabetic nephropathy	2824310, 22107891, 25573423	PTZ, AUD, Other
Citalopram	Depression; panic disorder	21531632, 21962757, 22429158, 22578701	KA, PILO, PTZ
Dapsone	Leprosy; dermatitis herpetiformis	1817960, 7970237, 23729301	KA, Kin
Dextromethorphan	Pain; addiction; cough	1456842, 2079649, 2574061, 2666123, 2676564, 2806362, 3044591, 3374269, 3380326, 3768695, 8058587, 8094234, 8405092, 8856734, 9179861, 9187330, 10080248, 11182165, 12479976, 12586225, 15084442, 15723099	KA, PTZ, Electro, AUD, Kin, other
Diltiazem	Angina; hypertension	2272645, 7681002, 8152336, 22661180	KA, PTZ, Electro
Doxepin	Depression; pruritus	1456842, 19443935	PTZ, Electro, other
Fluoxetine	Depression; bulimia nervosa; obsessive-compulsive disorder	7999524, 8149989, 8384110, 8538363, 8816259, 9696406, 15680343, 16531634, 17215106, 23530452, 25754610	Electro, AUD, other
Isradipine	Hypertension	8118482, 9595291	Electro, AUD
Lovastatin	Hypercholesterolaemia	21224519, 23253428, 23352156	KA, AUD
Nicardipine	Angina; hypertension	7681002, 8152336, 8872866, 10608279, 11742591	KA, PTZ, Kin, other
Nifedipine	Angina; hypertension; Raynaud's phenomenon; premature labour	1628595, 1698518, 1747472, 1865996, 1946038, 2085727, 2272645, 2713089, 2744396, 7681002, 7694769, 8054599, 8118482, 8152336, 8474621, 8707372, 12126870, 12536054, 16573711, 20113637, 22661180, 22801414	KA, PTZ, Electro, AUD, Kin, other
Nimodipine	Subarachnoid haemorrhage	1628595, 1698518, 2272645, 2310938, 2463174, 2662221, 3784769, 7681002, 8152336, 8156970, 8156971, 8707372, 9389584, 9570719, 9689485, 10683952, 12372903, 12536054, 12539272, 15123017, 17193898, 17344939, 19761108, 23761887, 25225705, 25445375	KA, Pilo, PTZ, Electro, AUD, Kin, other
Orphenadrine	Parkinsonism	2624511, 19815957	PTZ, Electro
Pimozide	Schizophrenia	2272645, 6141554, 7875556	PTZ, Electro, AUD, other
Pioglitazone	Diabetes mellitus	20599832, 22436324, 27527983	PTZ, other
Quetiapine	Schizophrenia; mania; depression	21168466, 26188240	PTZ, AUD
Tamoxifen	Breast cancer; anovulatory infertility	12139106, 24903749	Electro, Kin
Thalidomide	Malignant disease; immunosuppression	17449064, 21592729, 24735834	PTZ, Kin

Supplementary table 13.

Top 20 drugs that are licensed for conditions other than epilepsy, but are predicted to be efficacious for GGE, and have published evidence of antiseizure efficacy from multiple published studies and in multiple animal models. We do not advise immediate use of these drugs for people with epilepsy, prior to any clinical trials. AUD: audiogenic; electro: maximal electroshock; Kin: kindling; PTZ: pentylenetetrazol. Drugs are listed in alphabetical order.

Cohort	All epilepsy	Focal	GGE	Controls
**UK Biobank**	7,006	-	-	179,763
**Japan Biobank**	612	145	283	176,694
**DECODE genetics**	3,762	405	1,342	335,389
**FinnGen**	10,354	5,922	1,160	332,143
**Total**	21,734	6,472	2,785	1,023,989

Supplementary table 14.
Sample sizes of the included Biobanks and deCODE genetics.

	Primary All epilepsy	Primary Focal	Primary GGE
**Biobank All epilepsy**	0.74 (0.106)	0.5525 (0.1781)	0.7036 (0.0879)
**Biobank Focal**	0.5835 (0.1596)	0.7637 (0.2505)	0.4331 (0.1275)
**Biobank Gen**	0.6231 (0.1434)	0.307 (0.2176)	0.6521 (0.1373)

Supplementary table 15.
Genetic correlations between our main GWAS and Biobank GWAS (including deCODE genetics). two-tailed P-values are shown, with standard errors in brackets.

	Missense	TWAS	SMR	MAGMA	PoPS	Brain expression	Knockout mouse	Monogenic epilepsy gene	Target of AED	brain-coX
Missense	1.000	0.158**	0.490**	0.327**	0.039	-0.028	0.129*	0.145*	0.110	0.012
TWAS	0.158**	1.000	.305**	0.296**	0.043	-0.128*	0.088	-0.001	-0.042	-0.037
SMR	0.490**	.305**	1.000	0.389**	-0.052	-0.046	0.086	0.035	-0.031	-0.059
MAGMA	0.327**	.296**	.389**	1.000	.261**	-0.015	0.124*	0.144*	0.053	0.090
PoPS	0.039	0.043	-0.052	0.261**	1.000	0.169**	0.132*	0.195**	0.110	0.225**
Brain expression	-0.028	-0.128*	-0.046	-0.015	0.169**	1.000	0.142*	0.181**	0.222**	0.346**
Knockout mouse	0.129*	0.088	0.086	0.124*	.132*	.142*	1.000	0.216**	0.242**	0.175**
Monogenic epilepsy gene	0.145*	-0.001	0.035	0.144*	.195**	.181**	0.216**	1.000	0.392**	0.214**
Target of AED	0.110	-0.042	-0.031	0.053	0.109	0.222**	0.242**	0.392**	1.000	0.243**
brain-coX	0.012	-0.037	-0.059	0.090	0.225**	0.346**	0.175**	0.214**	0.243**	1.000

Supplementary table 16.
*Pearson correlation coefficients for the ten biological gene prioritization criteria. * Correlation is significant at the 0.05 level (2-tailed). ** Correlation is significant at the 0.01 level (2-tailed).*

Supplementary Figures

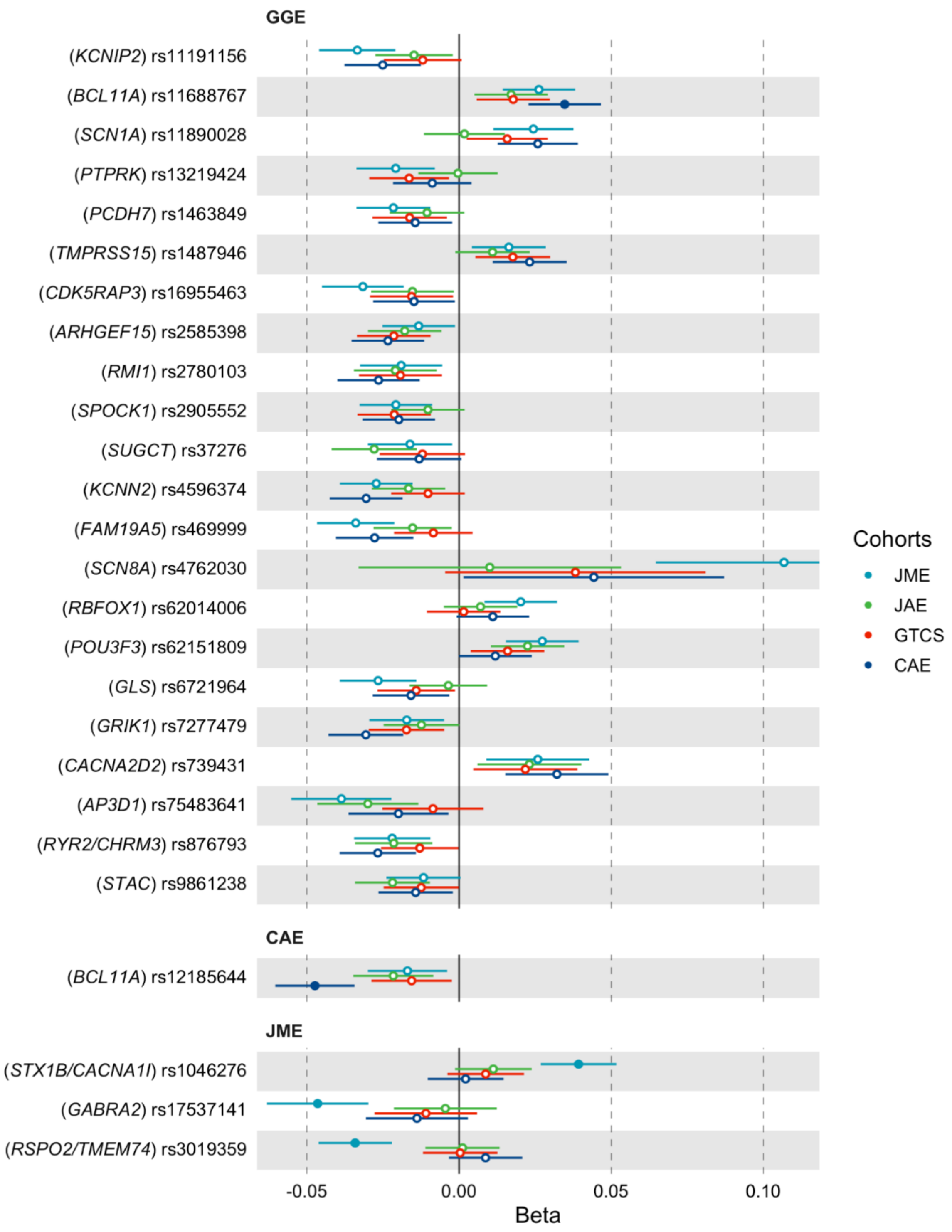

Supplementary figure 1.

Forest plots of the GGE (n=7407 cases in ILAE3), CAE (n=1072 cases in ILAE3) and JME (n=1813 cases in ILAE3) top hits. Beta effect estimates +/- s.e.m. are displayed for each of the 4 GGE subphenotypes. Genome-wide significant SNPs are displayed as filled points and non-significant as hollow points. X-axis limits are truncated between (-0.05,+0.10).

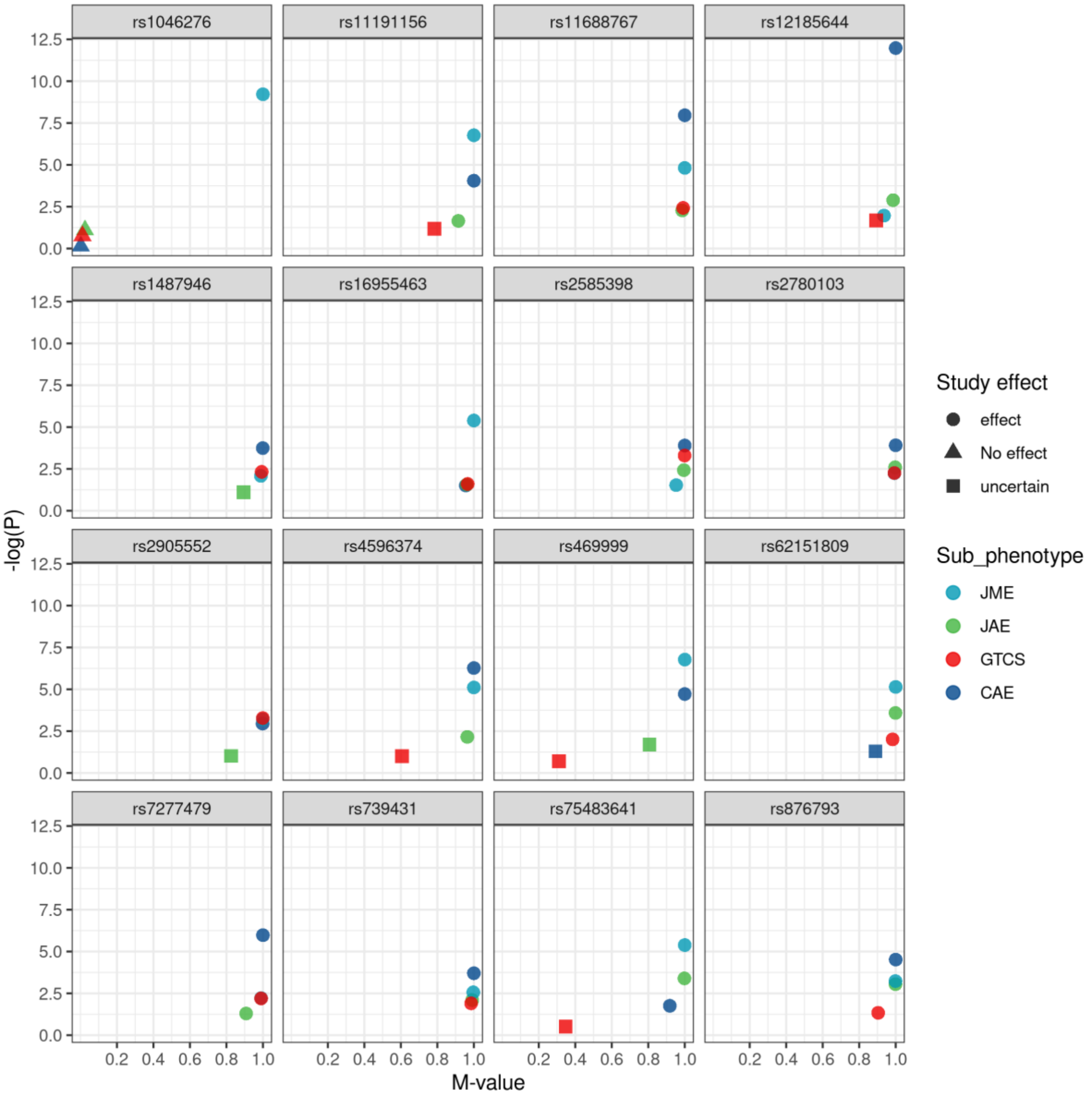

Supplementary figure 2.

PM-plots showing subphenotype specific two-sided P-value of the GGE, CAE and JME top hits against posterior probability m-value. Only SNPs with Meta two-sided P-value < 5×10−8 of subphenotypes were included. A study has an effect if m ≥ 0.9, uncertain effect if 0.1 < m < 0.9, and no effect if m ≤ 0.1. All values were calculated with MetaSoft PM analysis using default settings.

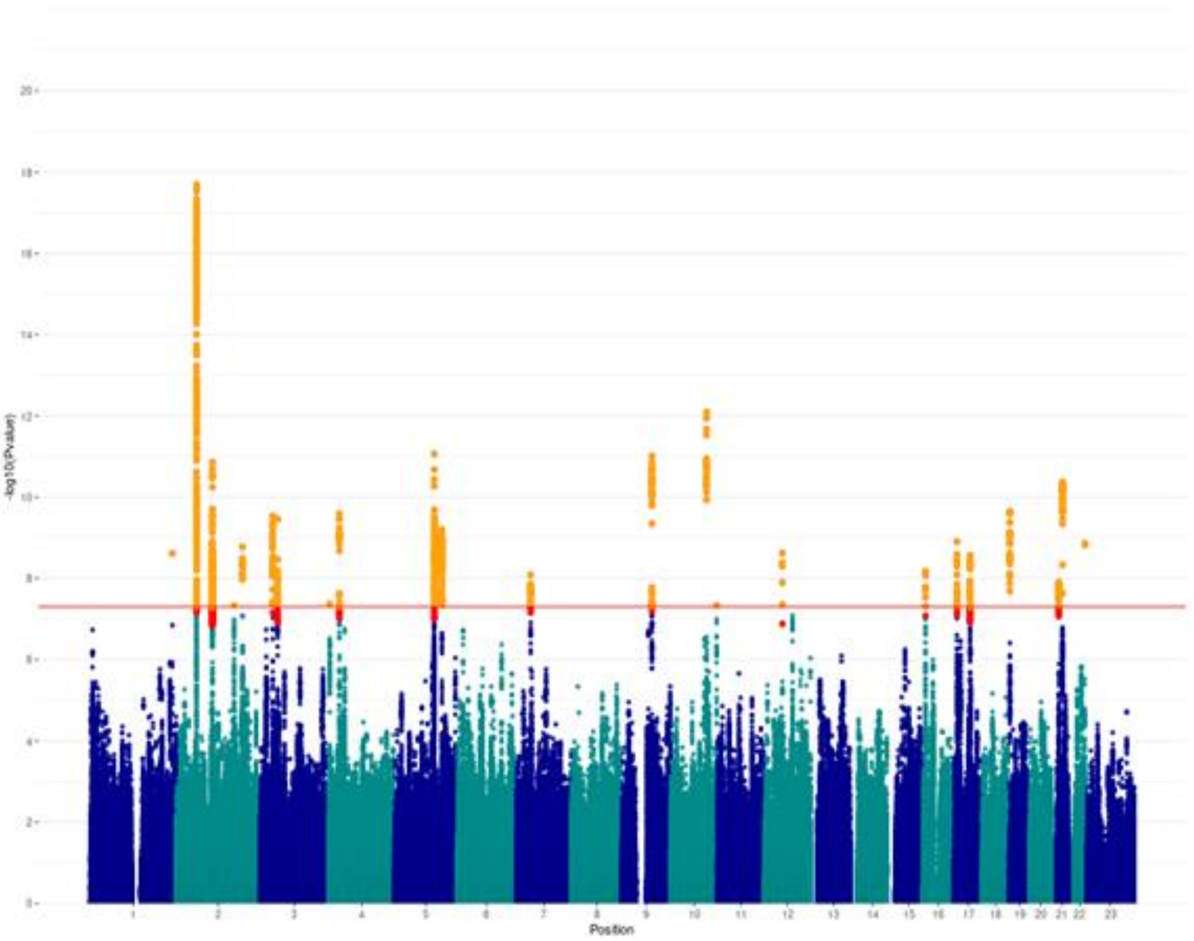

Supplementary figure 3.
Manhattan plot of MTAG significant variants for GGE, when combined with focal epilepsy, obtained with MTAG meta-analysis using default settings. The red dots are the previously significant SNPs in the meta-analysis that are no longer significant in MTAG. We did not identify any novel loci. Results are shown for GGE only because maxFDR value was sufficiently low for the GGE analysis (0.006). For FE this value was too high (0.6) suggesting that results for FE are likely spurious. The red line shows the genome-wide significance threshold (5x10-8). Chromosome and position are displayed on the x axis and two-sided -log10 P-value on the y axis.

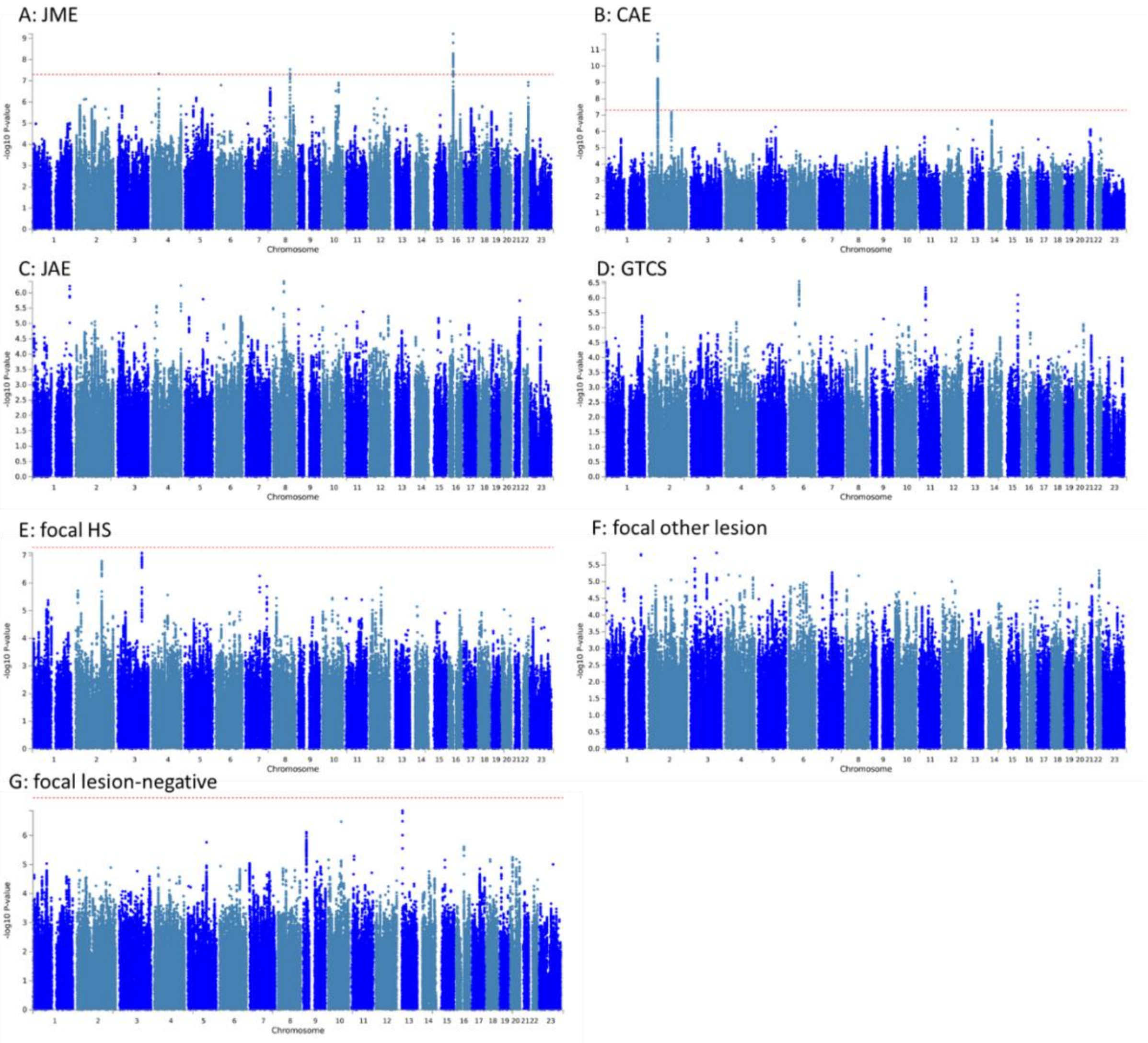

Supplementary figure 4.

Manhattan plots of epilepsy subphenotype GWAS. Chromosomal position is plotted on the X-axis and -log10 transformed P-values are plotted on the Y-axis, obtained by fixed-effects meta-analysis weighted by effective sample sizes. A. juvenile myoclonic epilepsy (JME); B. childhood absence epilepsy (CAE); C. juvenile absence epilepsy (JAE); D. generalized tonic-clonic seizures alone (GTCS); E. focal epilepsy due to hippocampal sclerosis (focal HS); F. focal epilepsy with other lesion; G. lesion negative focal epilepsy. The red line shows the genome-wide significance threshold (5x10-8). Chromosome and position are displayed on the x axis and two-sided -log10 P-value on the y axis.

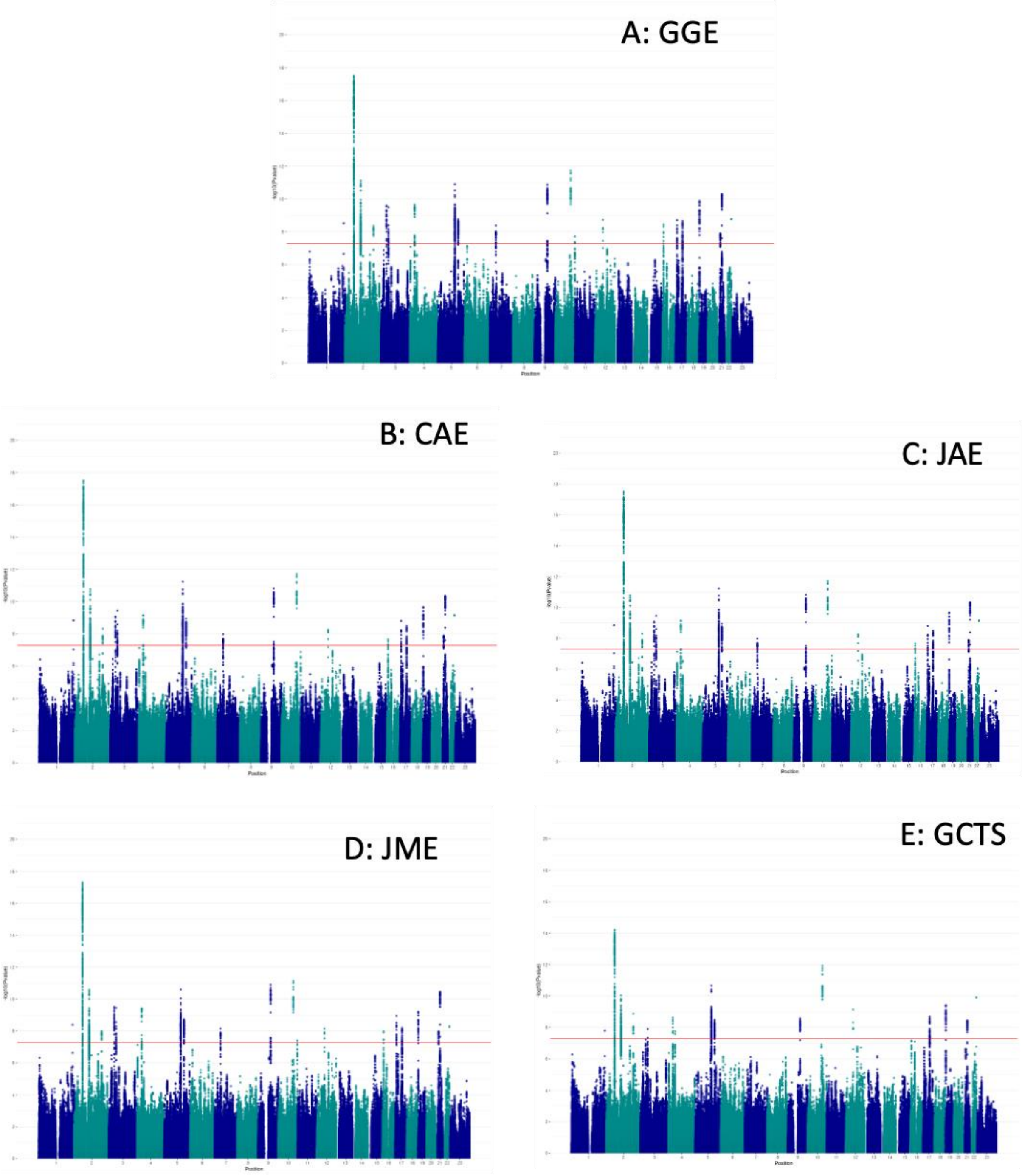

Supplementary figure 5.

GGE subphenotype MTAG meta-analysis: Manhattan plots. Chromosomal position is plotted on the X-axis and -log10 transformed two-tailed P-values are plotted on the Y-axis. A. genetic generalised epilepsy (GGE), ; B. childhood absence epilepsy (CAE); C. juvenile absence epilepsy (JAE); D. juvenile myoclonic epilepsy (JME); E. generalized tonic-clonic seizures alone (GTCS).

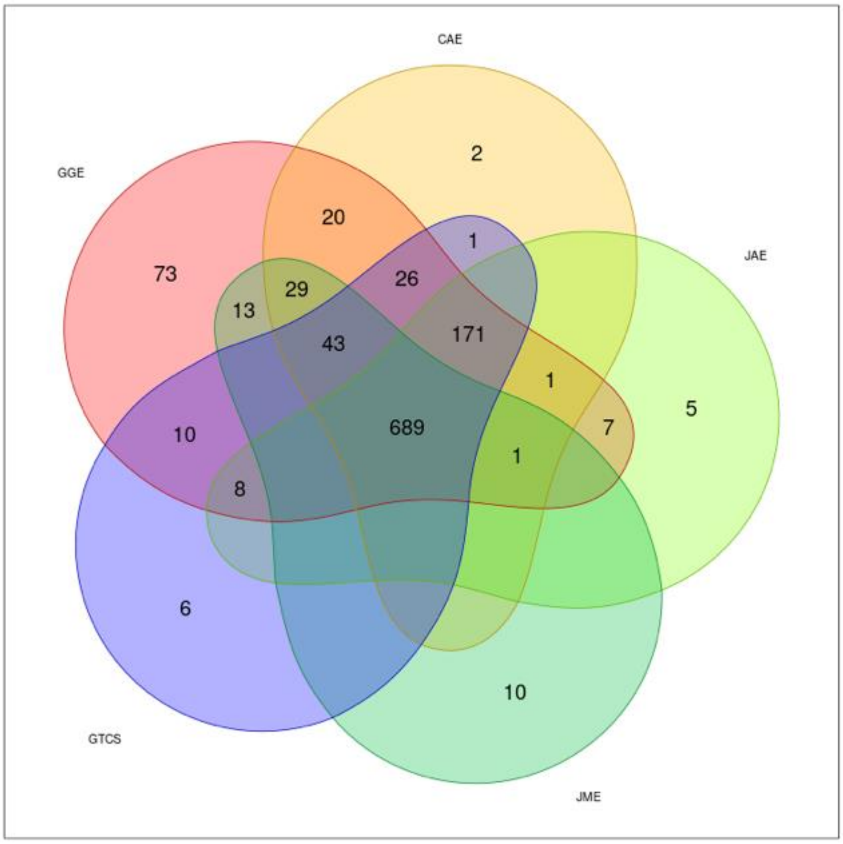

Supplementary figure 6.
MTAG analysis of GGE subphenotype shared GWAS significant SNPs. Figure shows the number of significant SNPs for each GGE sub-phenotype and their intersections.

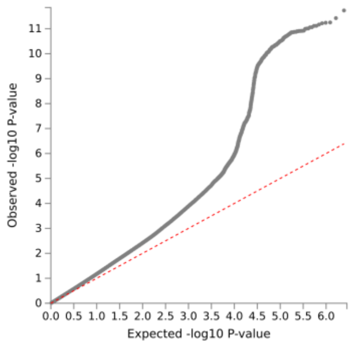	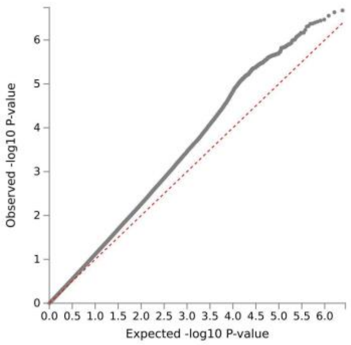	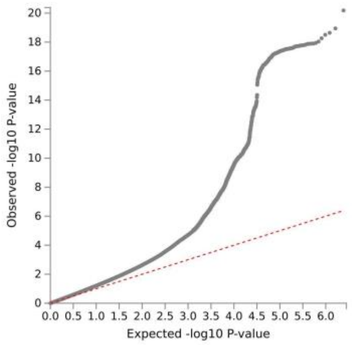
All epilepsy	*focal*	*GGE*

Supplementary figure 7.
Q-Q plots for 'all epilepsy', 'focal' and 'GGE' meta-analysis. Expected (X-axis) vs observed (Y-axis) -log10 two-tailed P-values are displayed.

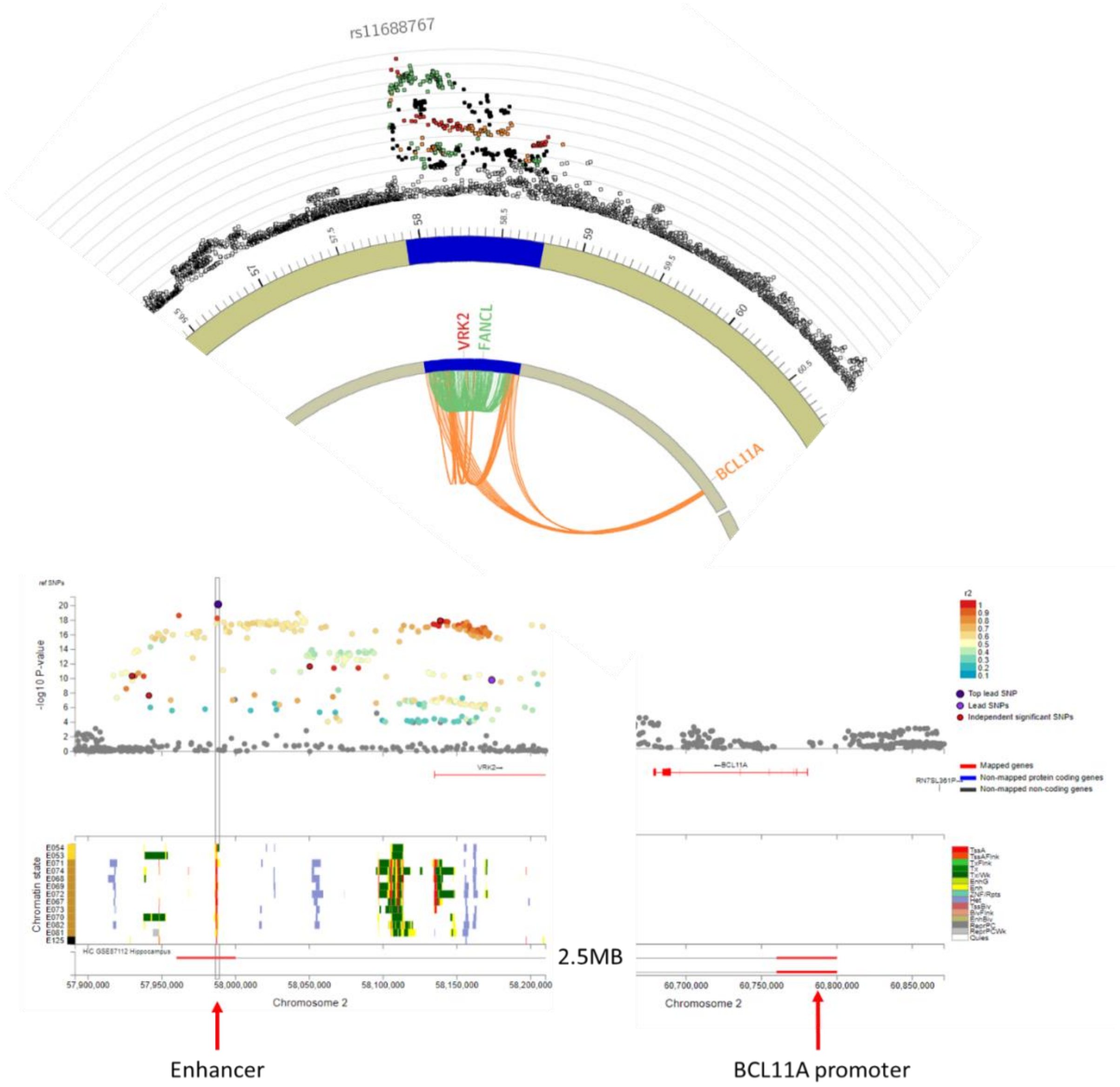

Supplementary figure 8.

3D chromatin interactions link the 2p16.1 locus with the promoter region of BCL11A. The upper circus plot shows the 2p16.1 locus with GWAS two-tailed P-values in the outer ring, with eQTL associations in green and HiC 3D chromatin interactions in orange. The locuszoom below shows GWAS two-tailed P-values with chromatin states and Hi-C chromatin interactions below.

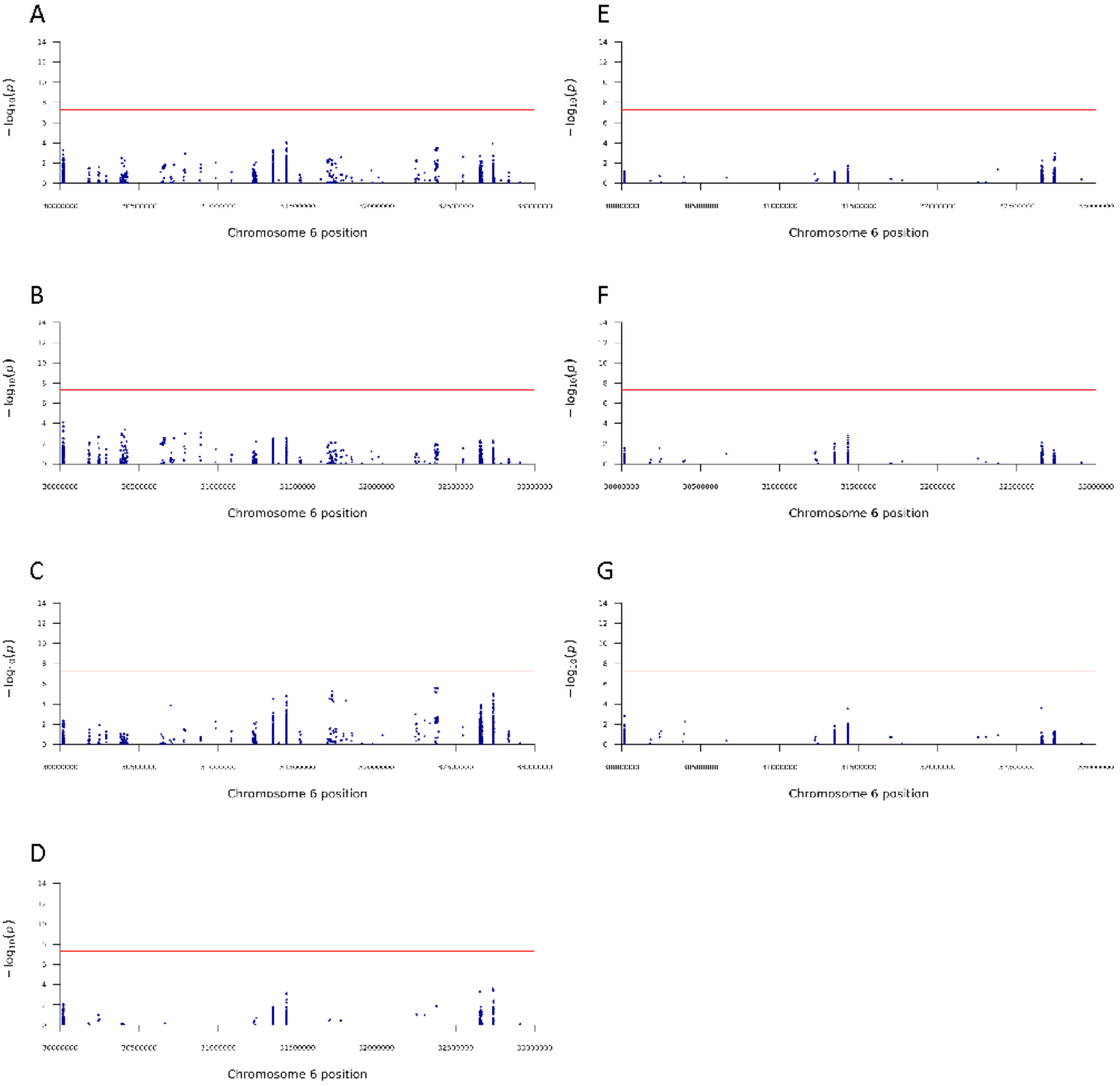

Supplementary figure 9.

Manhattan plots of HLA analysis for A) All Epilepsy, B) Focal Epilepsy, C) GGE, D) JME, E) Focal lesion negative, F) Focal due to other lesion, G) Focal HS, obtained by fixed-effects meta-analysis of HLA association tests The red line shows the genome-wide significance threshold (5x10-8). Chromosome and position are displayed on the x axis and two-sided -log10 P-value on the y axis.

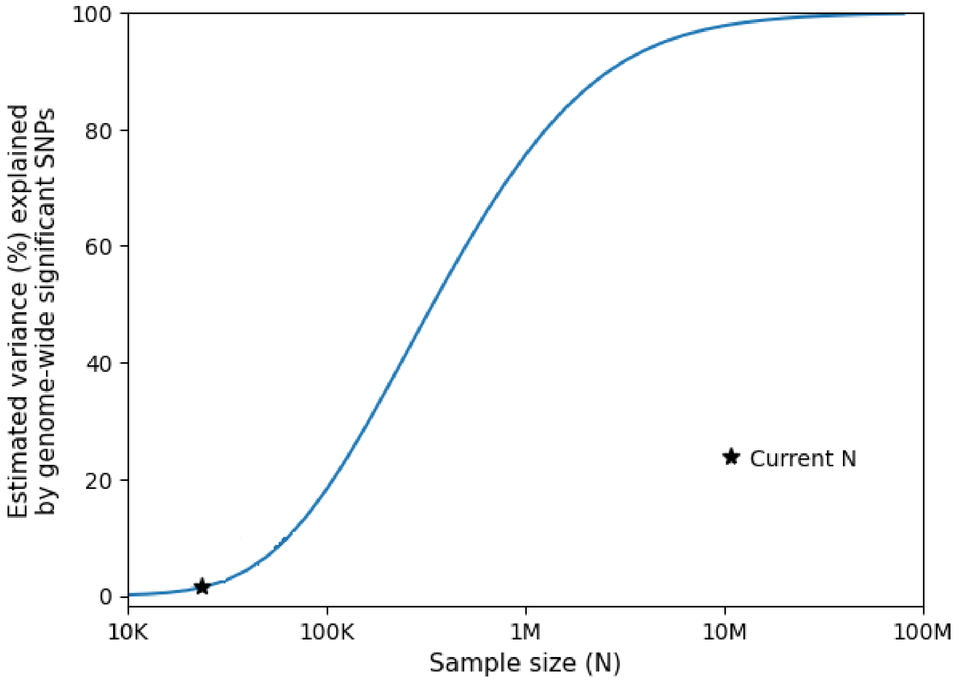

Supplementary figure 10.
Power analysis for GGE, using the MiXeR causal mixture model.[20] The X-axis shows the current and required sample size, and the Y-axis shows the corresponding explained variance by genome-wide significant SNPs at these sample sizes. An explained variance of 100% corresponds to the identification of all SNPs that underlie GGE SNP-based heritability.

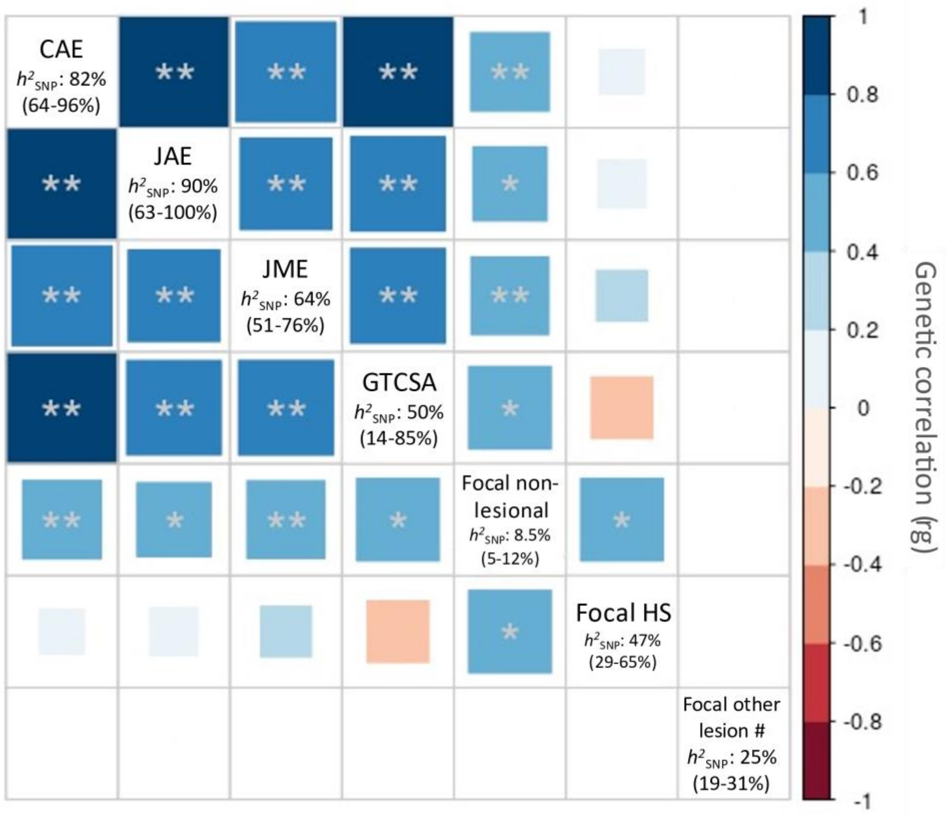

Supplementary figure 11.
*Heritability estimates and genetic correlations between epilepsy syndromes. Heritability measures were calculated using LDAK. The genetic correlation coefficient was calculated with LDSC and is denoted by color scale from -1 (red) to +1 (blue). # rg out of bounds due to phenotype not reaching significant heritability in LDSC; * Two-sided P < 0.05, ** Two-sided P < 0.0024 (Bonferroni correction).*

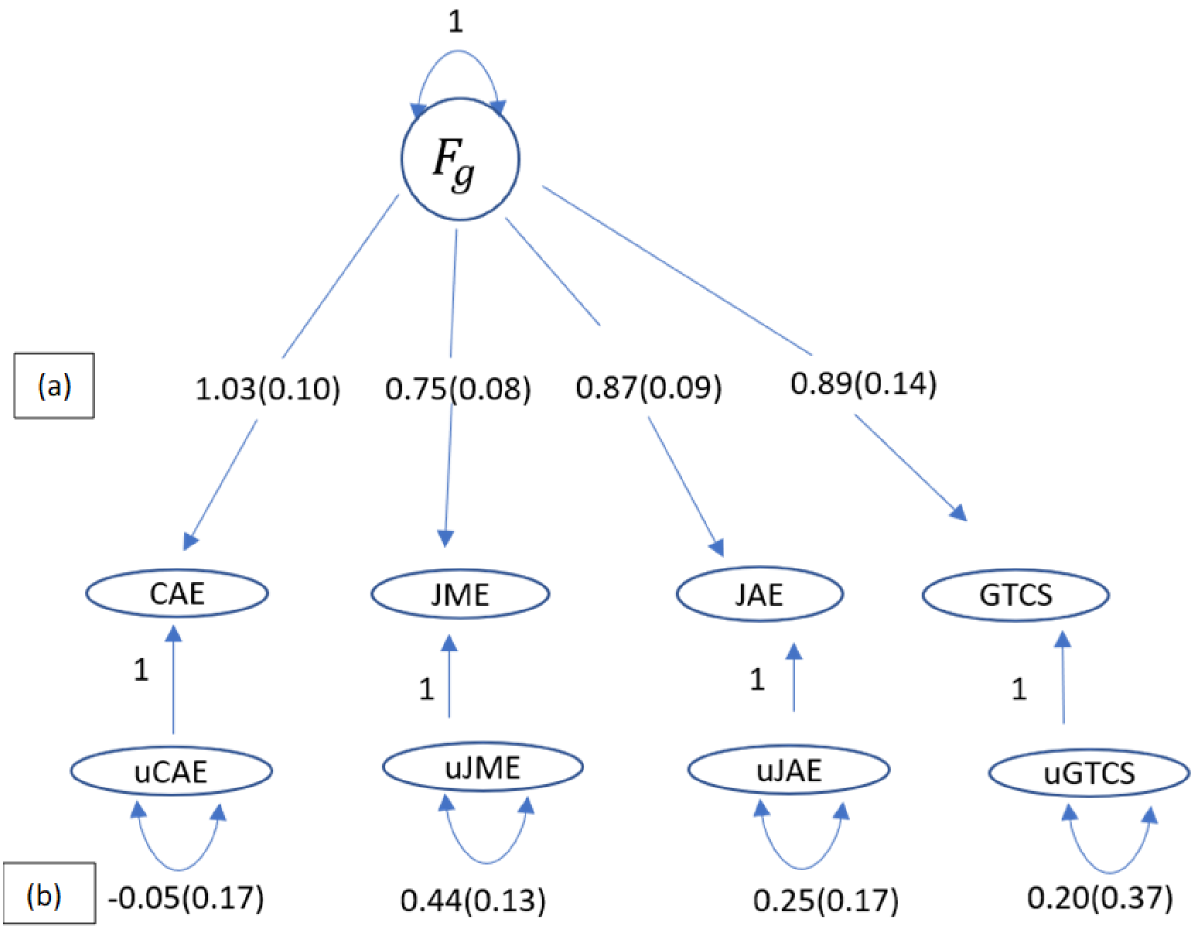

Supplementary figure 12.
Path diagram of confirmatory factor analysis model without SNP effects showing, (a) – standardized results of indicator loadings (genetic correlation) with their standard errors in the bracket and (b) – standardized residual variances of the indicators without the variance explained by common factors. Model fit statistics for the GGE subset only analysis is 0.50, AIC = 16.5.

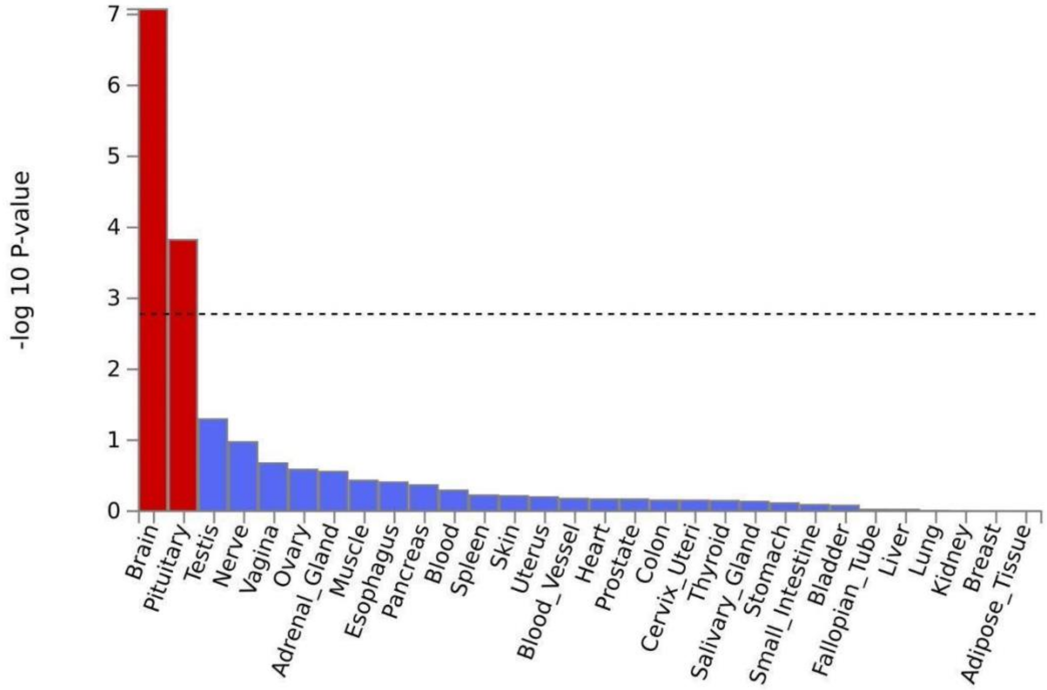

Supplementary figure 13.
Tissue-type enrichment of broad tissue types, as calculated with MAGMA tissue specificity analyses,[21] using data from the Gene-Tissue Expression consortium (GTEx). -log two-sided P-values are displayed on the Y-axis. The dotted line represents the significance threshold after correction for multiple comparisons and significant tests are shaded in red.

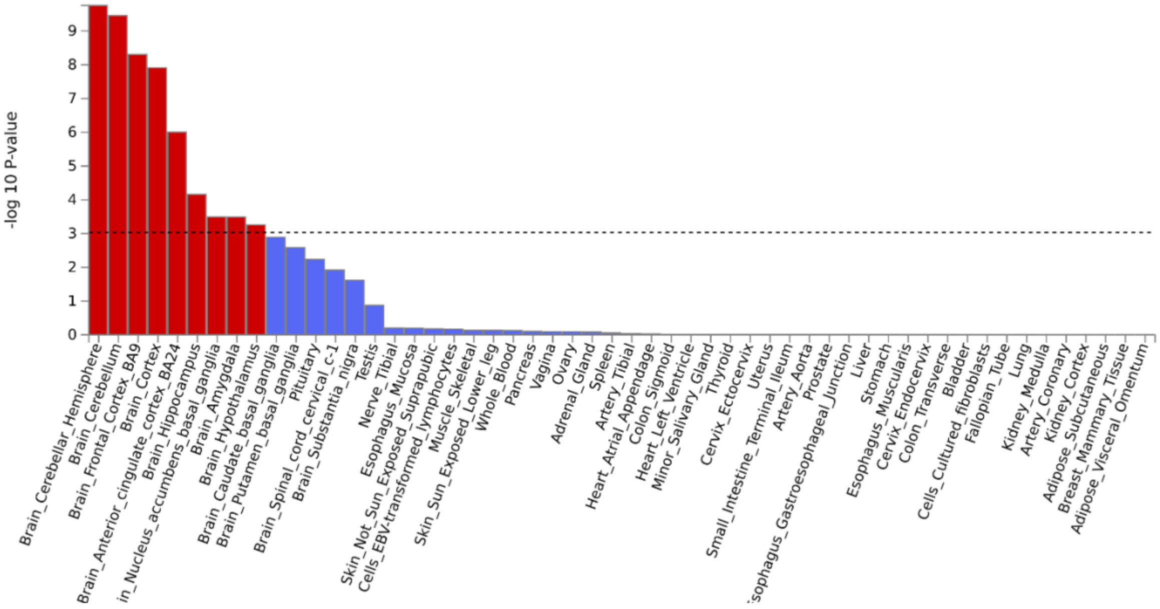

Supplementary figure 14.
Tissue-type enrichment of 54 tissues, including specific brain regions, as calculated with MAGMA tissue specificity analyses[21] using data from GTEx. -log two-sided P-values are displayed on the Y-axis. The dotted line represents the significance threshold after correction for multiple comparisons and significant tests are shaded in red.

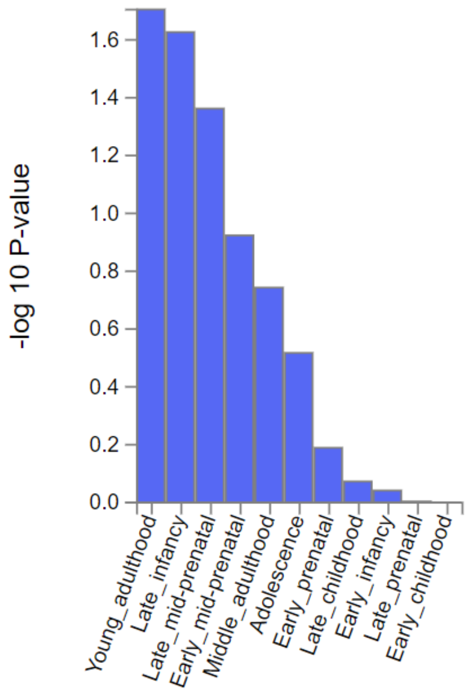

Supplementary figure 15.
Enrichment of genes expressed in the brain at 11 general developmental stages, as calculated with MAGMA tissue specificity analyses,[21] using data from the BrainSpan consortium. -log two-sided P-values are displayed on the Y-axis. None of the analyses were significant after correction for multiple comparisons.

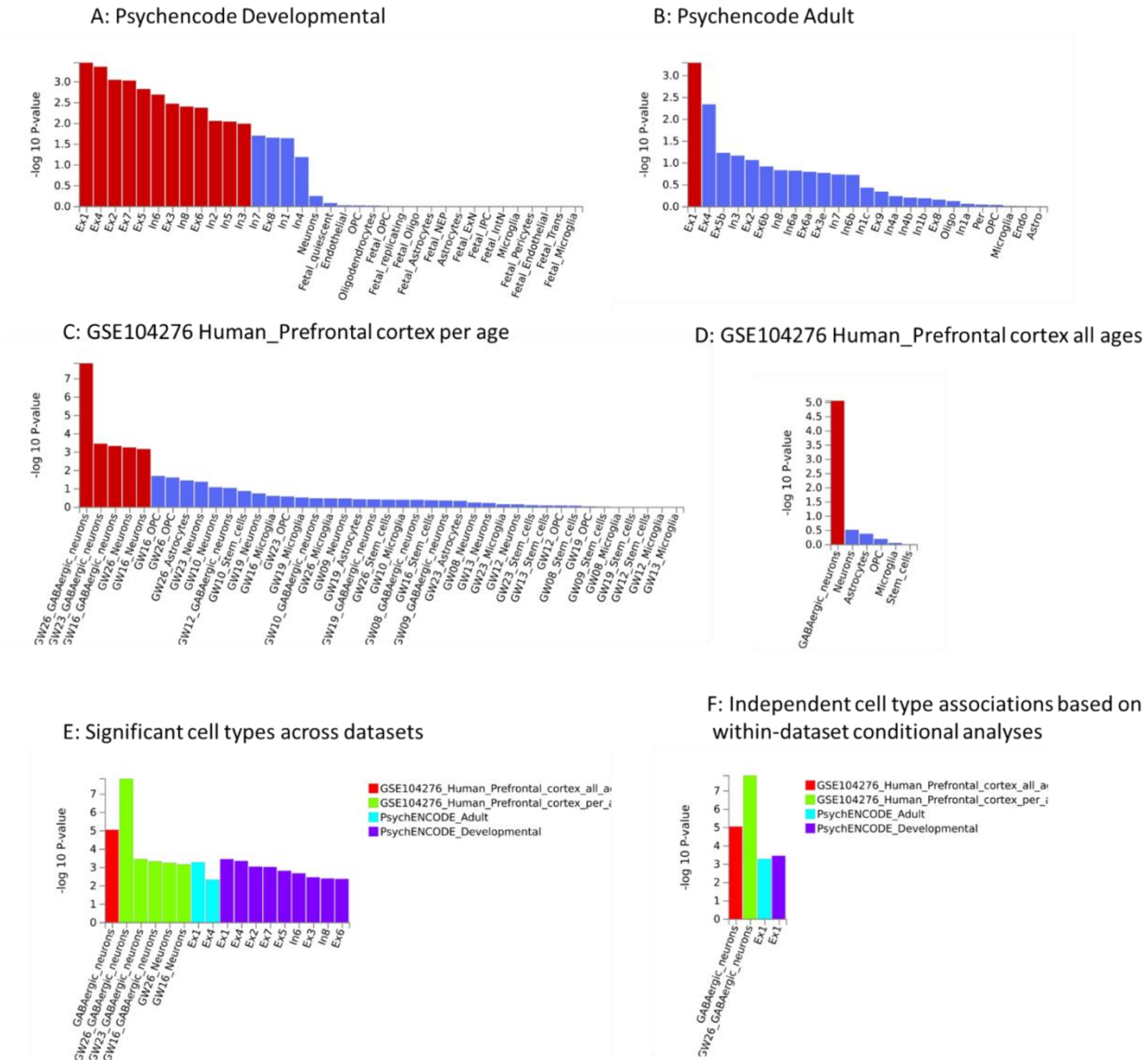

Supplementary figure 16.

Cell-type enrichment analyses across datasets, as calculated with FUMA cell specificity analyses.[22] Two different single-cell RNA sequencing datasets of human adult and developmental brain cells were assessed. Results from individual datasets are displayed in A-D with significant associations (after FDR multiple-comparison correction) shaded in red. -log two-sided P-values are displayed on the Y-axis. Significant cell types across datasets are displayed in E, and significant cell-types after within dataset conditional analyses (corrected for multiple comparisons) are displayed in F. Ex: excitatory neuron; In: inhibitory neuron.

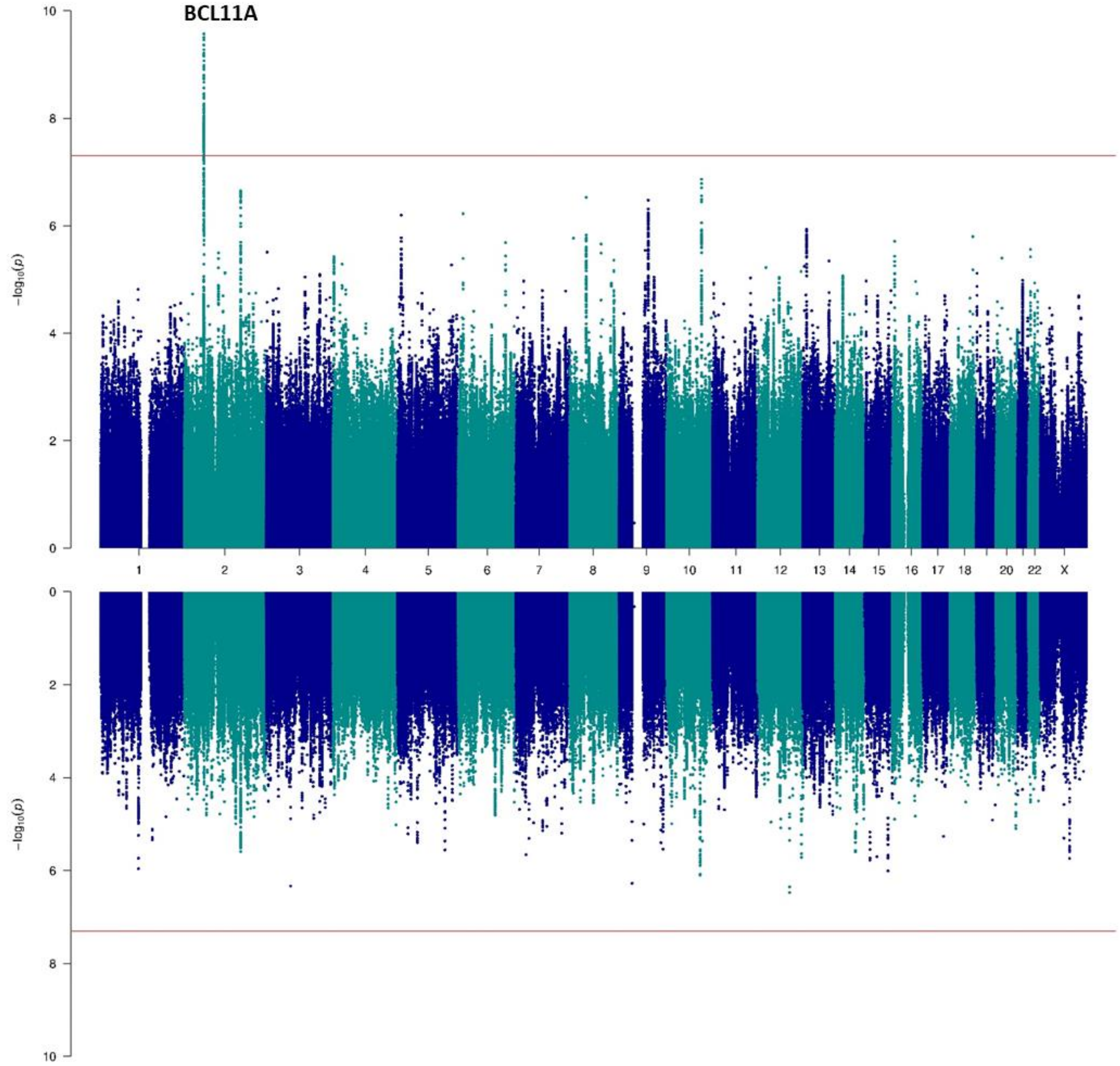

Supplementary figure 17.
Sex-specific GWAS of all epilepsy, obtained by fixed-effects meta-analysis weighted by effective sample sizes. The female-only is displayed at the top (n=13889 cases and 19676 controls) and male-only GWAS is displayed at the bottom (n=12259 cases and 18645 controls). We annotated genes that were implicated by our gene prioritization analyses. The red line shows the genome-wide significance threshold (5x10-8). Chromosome and position are displayed on the x axis and two-sided -log10 P-value on the y axis.

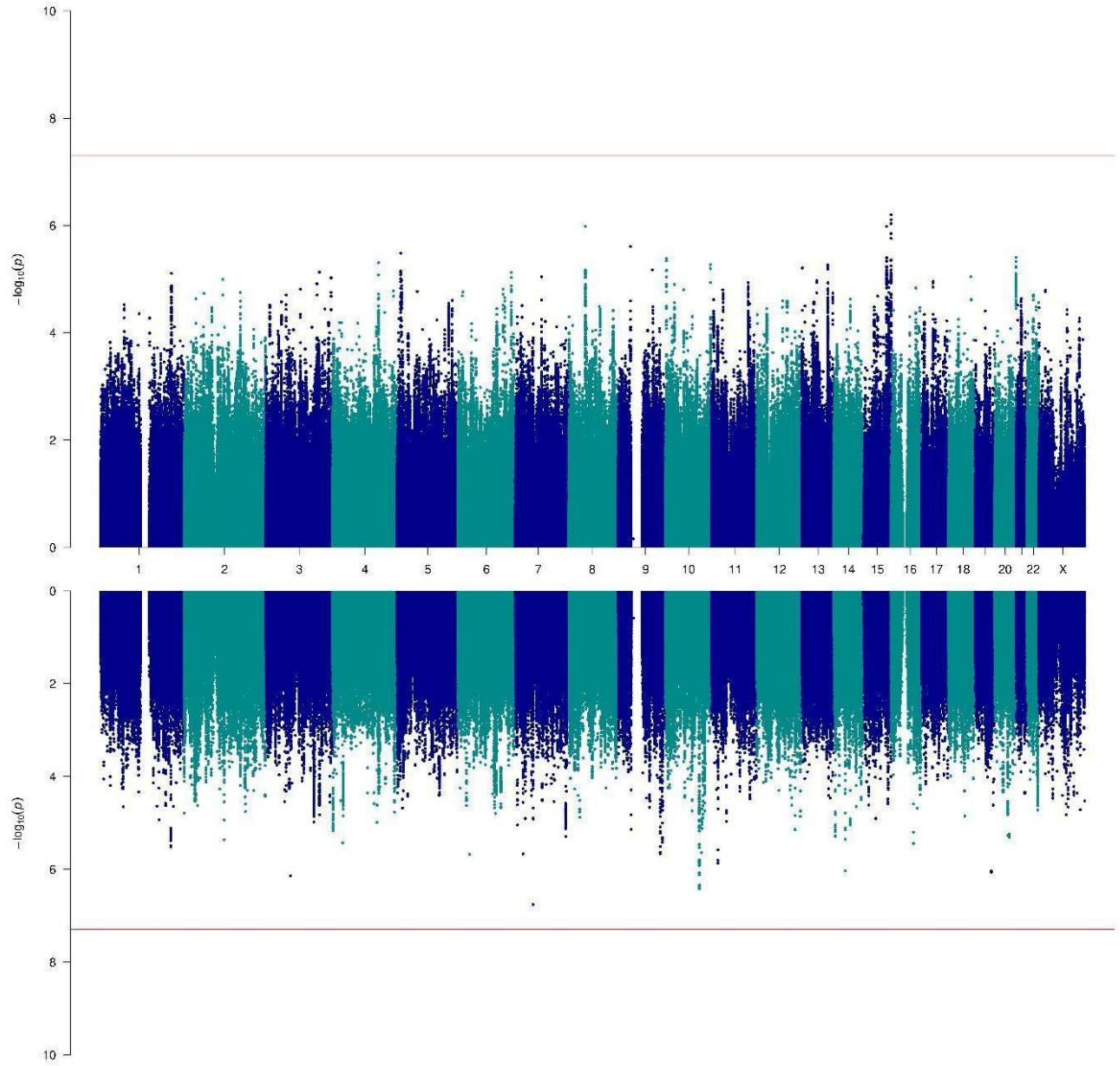

Supplementary figure 18.
Sex-specific GWAS of focal epilepsy, obtained by fixed-effects meta-analysis weighted by effective sample sizes. The female-only is displayed at the top (n=7175 cases and 19676 controls) and male-only GWAS is displayed at the bottom (n=6756 cases and 18645 controls). The red line shows the genome-wide significance threshold (5x10-8). Chromosome and position are displayed on the x axis and two-sided -log10 P-value on the y axis.

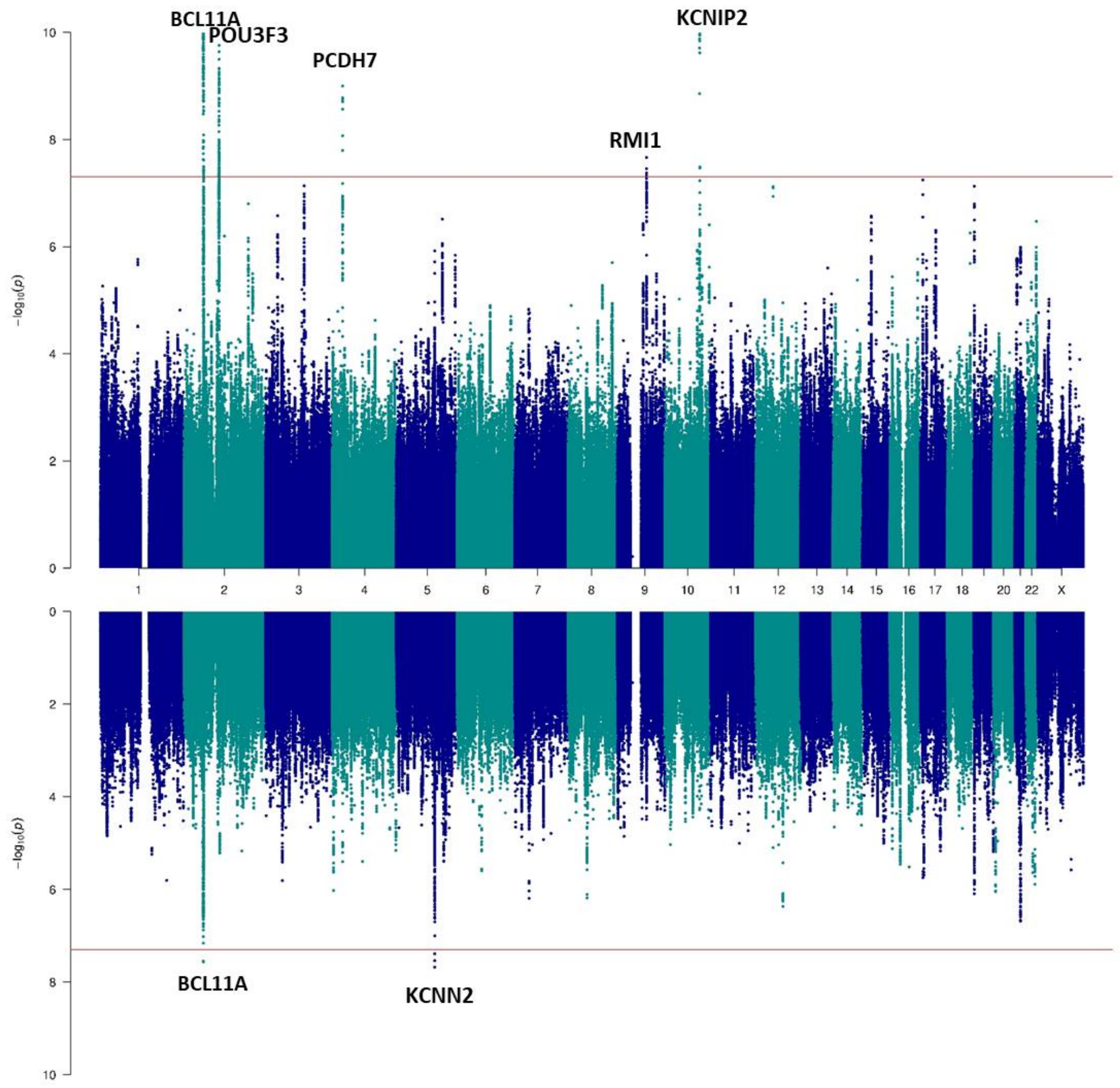

Supplementary figure 19.

Sex-specific GWAS of GGE, obtained by fixed-effects meta-analysis weighted by effective sample sizes. The female-only is displayed at the top (n=3946 cases and 19676 controls) and male-only GWAS is displayed at the bottom (n=2603 cases and 18645 controls). We annotated genes that were implicated by our gene prioritization analyses. The red line shows the genome-wide significance threshold (5x10-8). Chromosome and position are displayed on the x axis and two-sided -log10 P-value on the y axis.

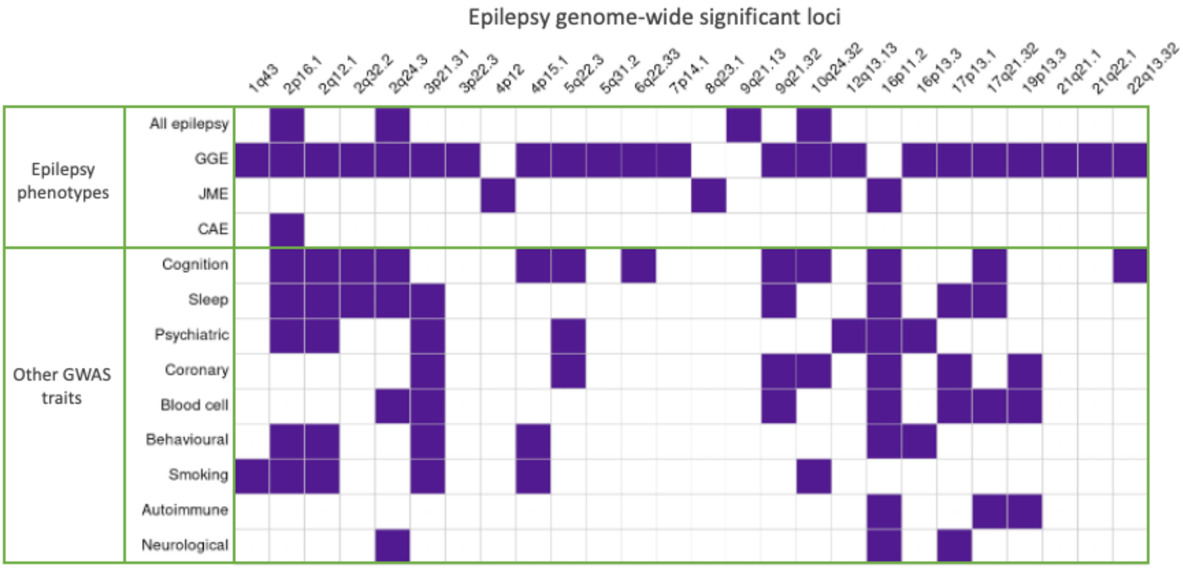

Supplementary figure 20.

*GWAS traits each of the epilepsy genome-wide significant loci have been associated with indicated by a purple cell. Prior trait associations were determined by a two-tailed P < 5*10-8 GWAS Catalog entry for the same SNP, or SNPs in high LD, as those reported in the epilepsy analysis.*

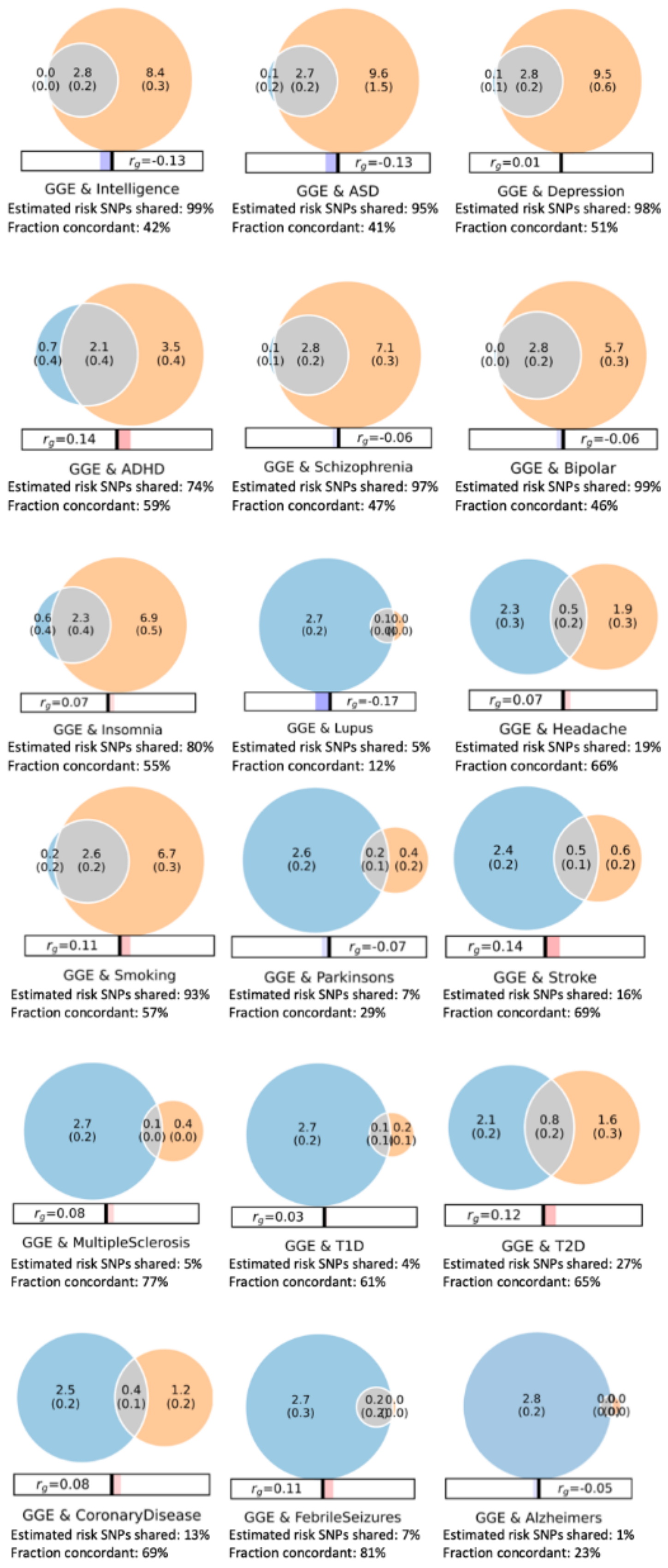

Supplementary figure 21.
Bivariate MiXeR analyses[20] showing the fraction of causal SNPs that are unique to GGE (blue), and shared (grey) and unique to 18 selected traits. Rg: genetic correlation coefficient.

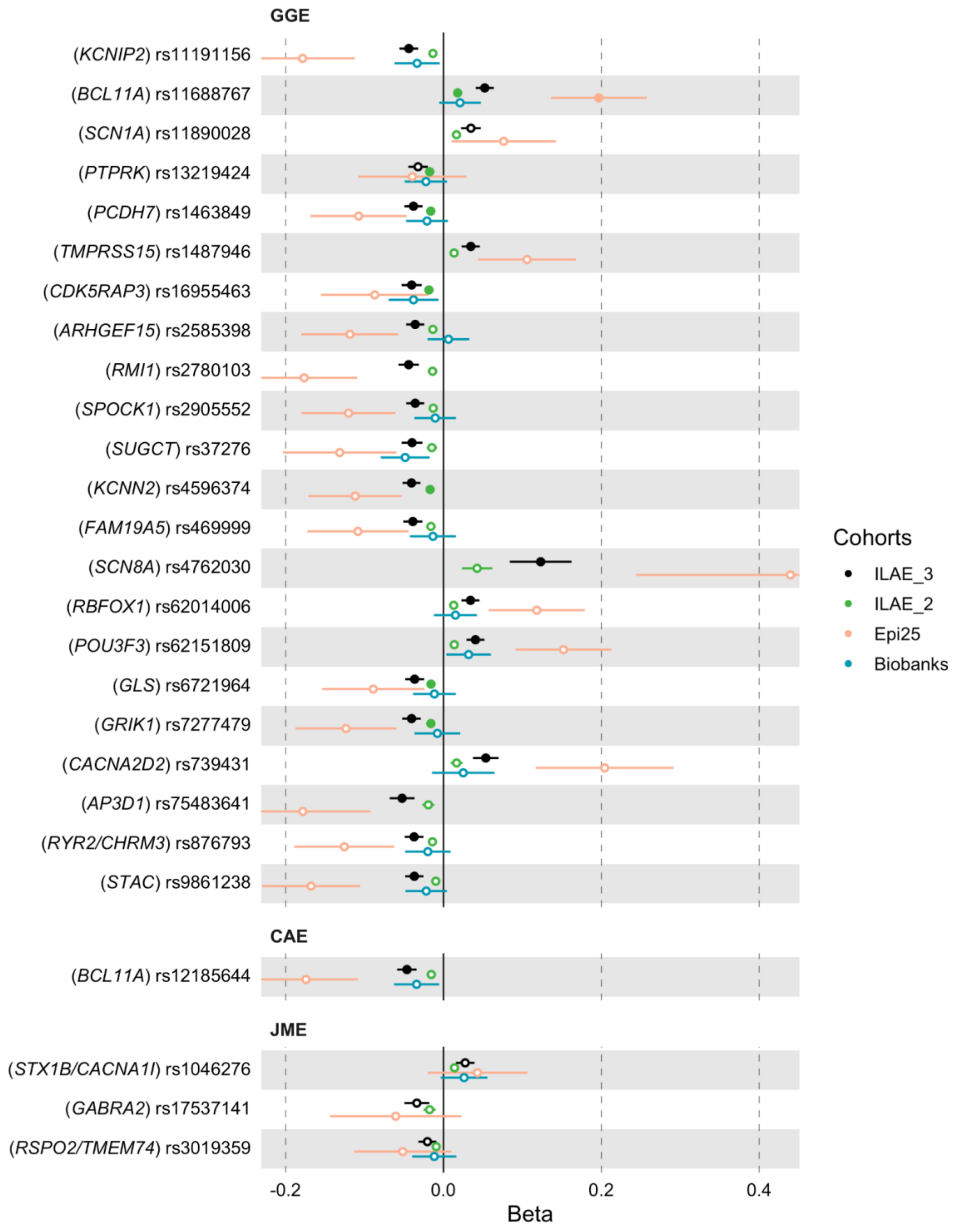

Supplementary figure 22.

Forest plots of the GGE (n=7407 cases in ILAE3), CAE (n=1072 cases in ILAE3) and JME (n=1813 cases in ILAE3)) top hits. Beta effect estimates showing effect sizes +/- s.e.m. are displayed for each of the four sumstats. Genome-wide significant SNPs are displayed as filled points and non-significant as hollow points. X-axis limits are truncated between (-0.2,+0.4). The effect sizes of 7 SNPs are not shown for the Biobank cohort due to missing genotype data. The magnitude of effect sizes cannot be directly compared between the different cohorts due to scaling differences. For ILAE2 and ILAE3 meta-analysis, beta and SE values were calculated from Z-scores and P-values as described previously.[23] The scaling of this conversion does not directly match the beta and SE values of the Epi25 and Biobank GWAS.

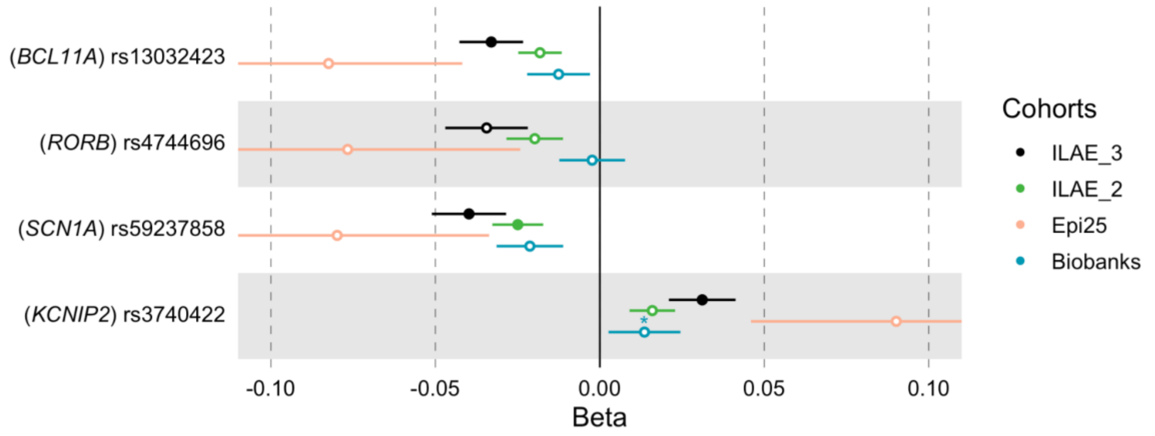

Supplementary figure 23.

Forest plots of the all epilepsy (n=29,944 cases in ILAE3) top hits. Beta effect estimates showing effect sizes +/- s.e.m. are displayed for each of the four cohorts included in this study. Genome-wide significant SNPs are displayed as filled points and non-significant as hollow points. X-axis limits are truncated between (-0.10,+0.10). "ILAE2 = results in the consortium cohort previously published [4], "Epi25" = results in the unpublished GWAS cohort from the Epi25 collaborative[10] (see Supplementary Table 1), "ILAE3" = results in the four additional cohorts included in the overall meta-analysis(see Supplementary Table 1), "biobanks" = results for all biobank cohorts combined as described in Supplementary Table 10. Asterisk reports that values for this SNP are from UK biobank and Finngen meta-analysis (It is not present in deCODE and Biobank Japan). The magnitude of effect sizes cannot be directly compared between the different cohorts due to scaling differences. For ILAE2 and ILAE3 meta-analysis, beta and SE values were calculated from Z-scores and P-values as described previously.[23] The scaling of this conversion does not directly match the beta and SE values of the Epi25 and Biobank GWAS.

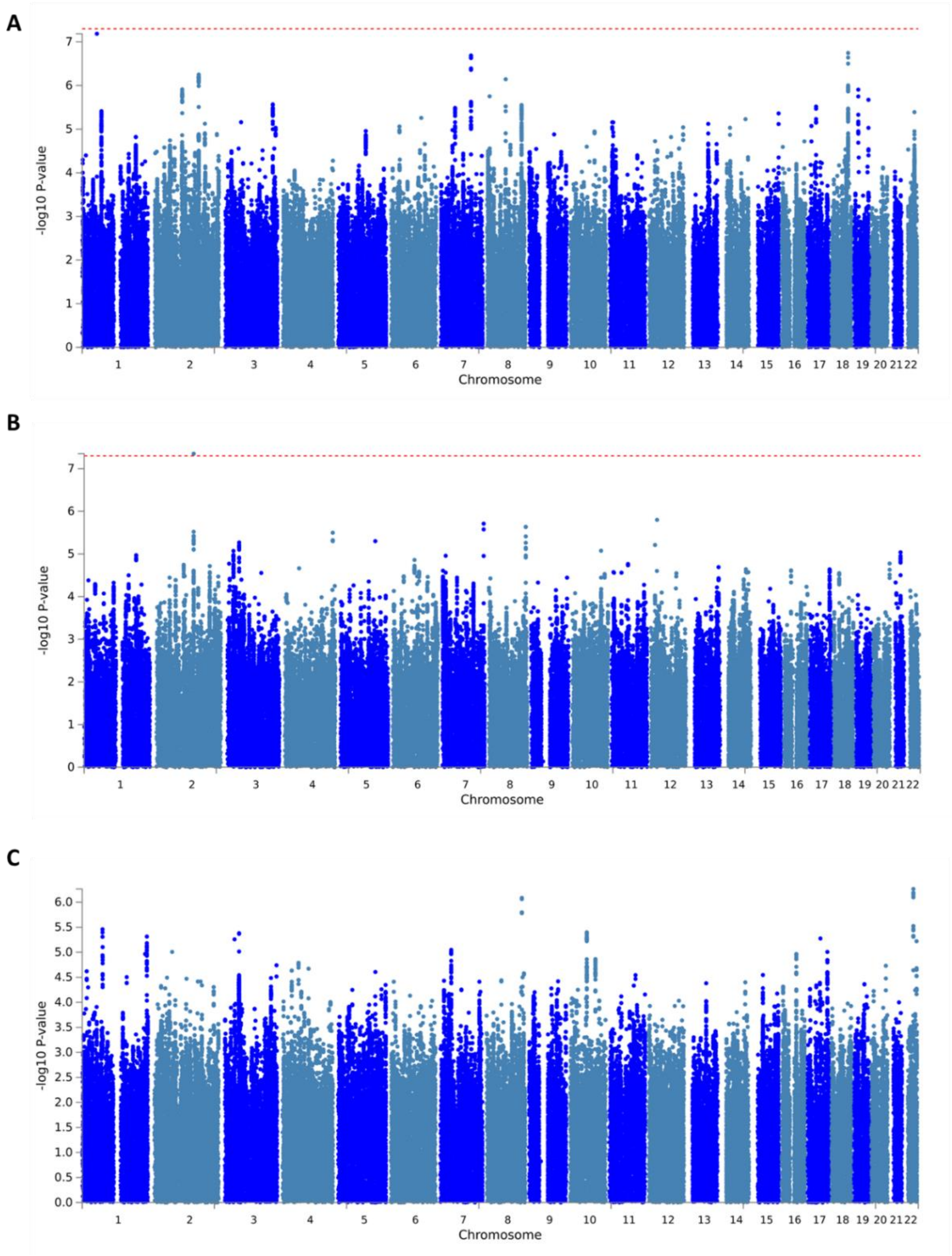

Supplementary figure 24.

Manhattan plots of Biobank-only GWAS of all (A), focal (B) and GGE (C), obtained by fixed-effects meta-analysis weighted by effective sample sizes. Chromosomal position is plotted on the X-axis and -log10 transformed two-sided P-values are plotted on the Y-axis.

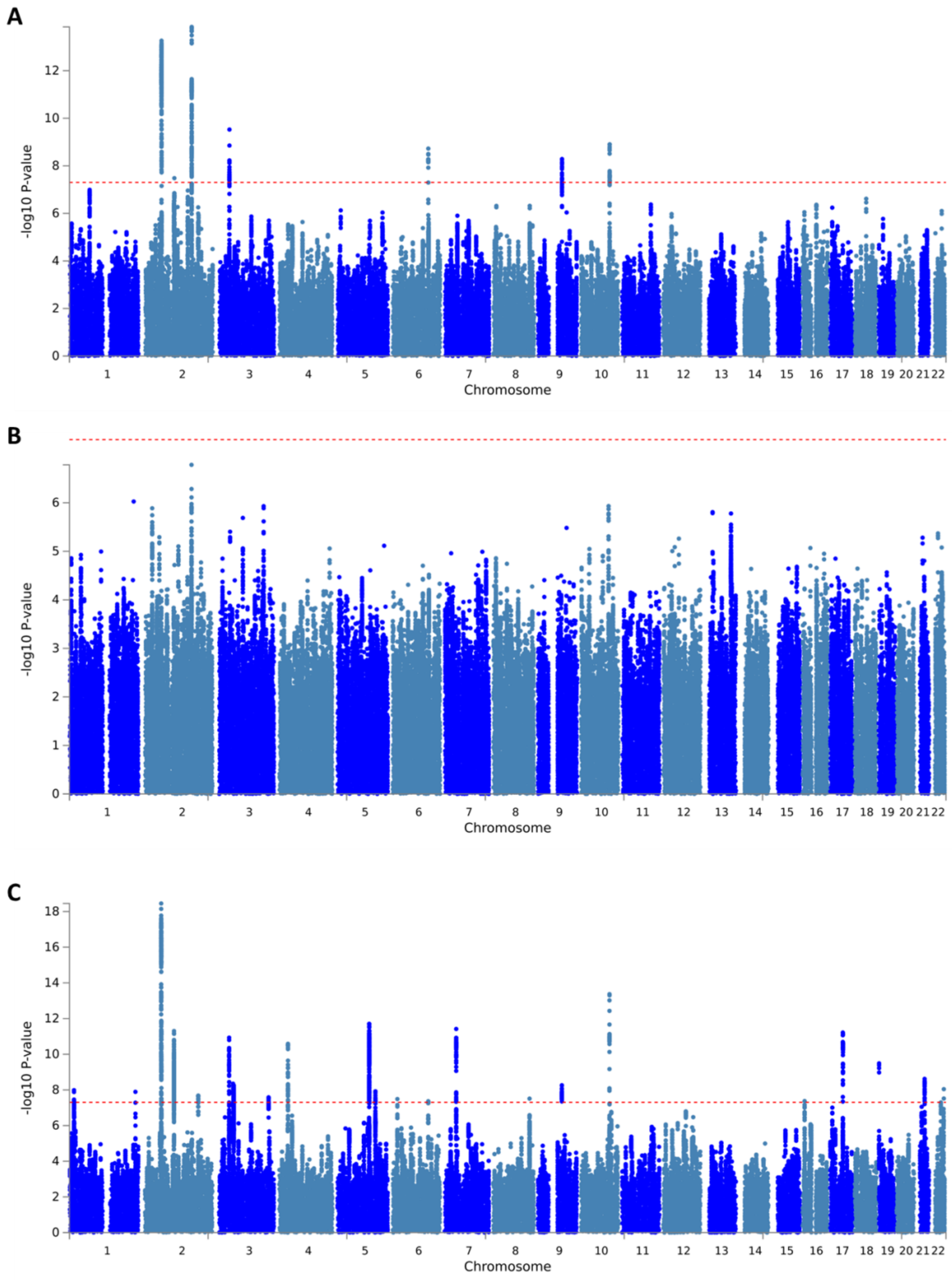

Supplementary figure 25.

Manhattan plots of meta-analysis combining the Biobanks with our primary GWAS of all (A), focal (B) and GGE (C), obtained by fixed-effects meta-analysis weighted by effective sample sizes. Chromosomal position is plotted on the X-axis and -log10 transformed two-sided P-values are plotted on the Y-axis.

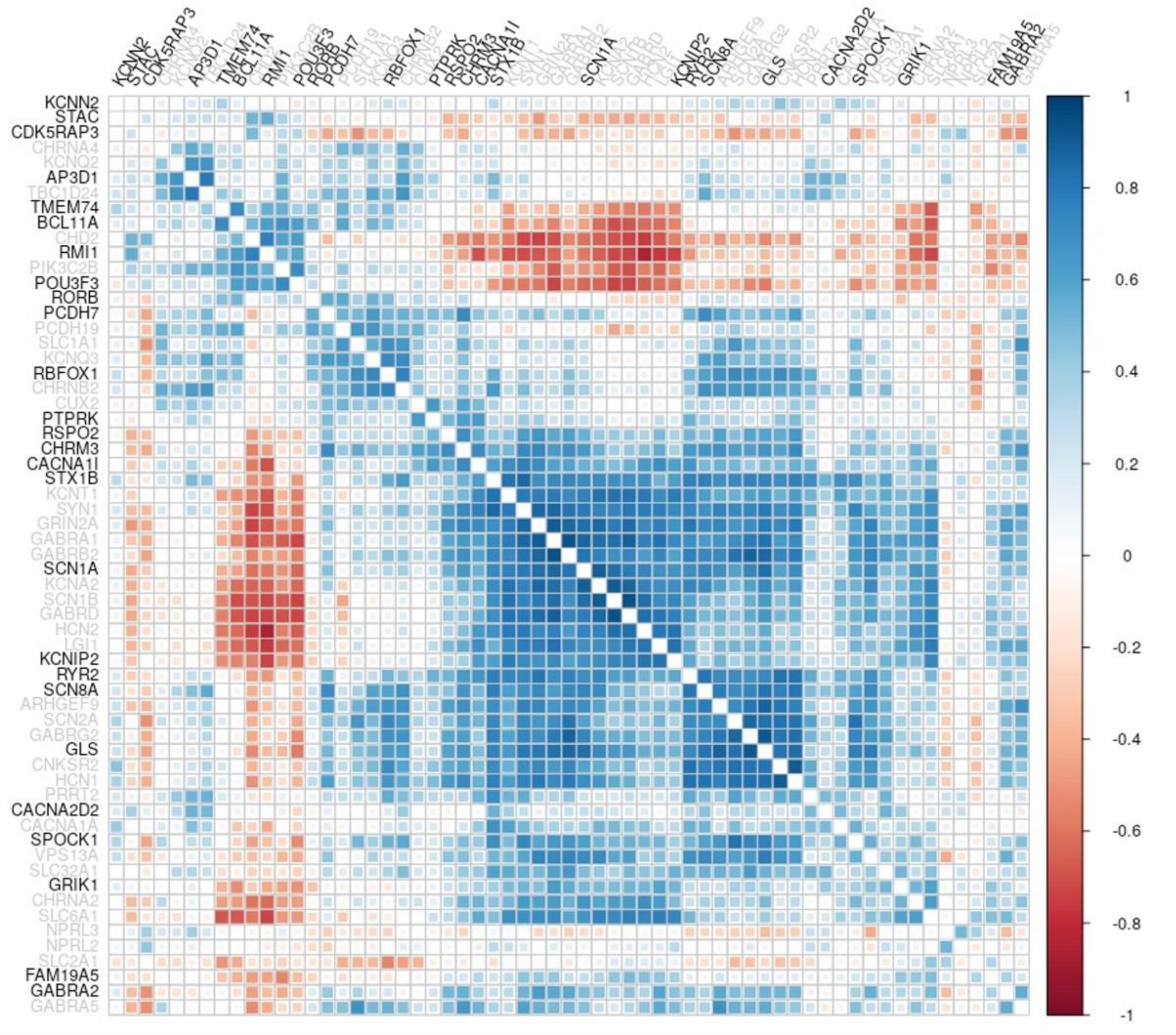

Supplementary figure 26.

Gene co-expression matrix produced by brain-coX[24] for known (grey) and candidate (black) epilepsy genes.

Supplementary Note

Summary of external control datasets used in the Epi25 GWAS

FINRISK controls

Description:

The controls from FINRISK that contributed to the Epi25 GWAS study were part of the FINRISK inflammatory bowel disease (IBD) cohort. The population-based FINRISK study has been followed up for IBD and other disease end-points using annual record linkage with the Finnish National Hospital Discharge Register, the National Causes-of-Death Register and the National Drug Reimbursement Register. Controls were chosen to have a high polygenic risk score for IBD without an IBD diagnosis. A detailed description of the FINRISK cohort can be found at Borodulin et al *(Borodulin, K., Tolonen, H., Jousilahti, P., Jula, A., Juolevi, A., Koskinen, S., Kuulasmaa, K., Laatikainen, T., Mannisto, S., Peltonen, M., et al. (2017). Cohort Profile: The National FINRISK Study. Int J Epidemiol.)*

Acknowledgements/Funding:

The FINRISK controls were part of the FINRISK studies supported by THL (formerly KTL: National Public Health Institute) through budgetary funds from the government, with additional funding from institutions such as the Academy of Finland, the European Union, ministries and national and international foundations and societies to support specific research purposes. Genotyping of FINRISK controls was supported by the Stanley Center for Psychiatric Research, Broad Institute, Cambridge, MA. GSA data are available via an application through the THL Biobank portal https://thl-biobank.elixir-finland.org/

Genomic Psychiatry Cohort (GPC)

Description:

The controls from GPC that were contributed to Epi25 study were a subset of the overall control participants with no personal or family history of schizophrenia or bipolar disorder. All the samples were genotyped on the GSA-MD v.1.0 at the Broad Institute. A detailed description of the GPC cohort can be found at Pato et al *(Pato, M.T., Sobell, J.L., Medeiros, H., Abbott, C., Sklar, B.M., Buckley, P.F., Bromet, E.J., Escamilla, M.A., Fanous, A.H., Lehrer, D.S., et al. (2013). The genomic psychiatry cohort: partners in discovery. Am J Med Genet B Neuropsychiatr Genet 162B, 306-312.)*

Acknowledgements/funding:

The GPC controls were genotyped on the GSA-MD v1.0 by the Broad Genomics Platform with funding from NIH grant U01MH105641 and the Stanley Center for Psychiatric Research, Broad Institute of MIT and Harvard. Data availability: GSA-MD v1.0 data for GPC samples data will be made available in dbGAP/AnVIL under study phs002041.

Hong Kong Osteoporosis Study (HKOS)

Description:

The control samples were part of the follow-up study from the Hong Kong Osteoporosis Study (HKOS), which was described elsewhere (Cheung et al 2018). Briefly, community-dwelling Southern Chinese were firstly recruited from public roadshows in Hong Kong from 1995 to 2010. An extensive in-person follow-up study was initiated in 2015. At the in-person follow-up visit, the study participants were required to complete a comprehensive self-reported questionnaire, comprising questions related to their medical history, which were checked by experienced researchers or nurses based on a standard protocol. Fasting blood samples were collected from the study participants and DNA was extracted from the sera samples. Study participants without any history of epilepsy at the in-person follow-up in 2019 were included as controls of the epilepsy project. The study protocol was approved by the Institutional Review Board of the University of Hong Kong and the Hospital Authority Hong Kong West Cluster (Ref: UW 15-236). All HKOS participants provided informed consent for participation in the study. (*Cheung CL, Tan KCB, Kung AWC. Cohort Profile: The Hong Kong Osteoporosis Study and the follow-up study. Int J Epidemiol. 2018 Apr 1;47(2):397-398f. doi: 10.1093/ije/dyx172. PMID: 29024957.)*

Acknowledgements/funding:
The collection of samples was funded by the Bone Health Fund and Research Grants Council - Early Career Scheme (Project number: 27100416). Genotyping of samples on the GSA-MD v1 was done by the Broad Genomics Platform and supported by the NHGRI CCDG grant (1UM1HG008895). GSA data will be made available in dbGaP/AnVIL under phs001489.

NIDDK Inflammatory Bowel Disease Genetics Consortium (NIDDK IBDGC)

Description/Acknowledgements/funding:
The NIDDK Inflammatory Bowel Disease Genetics Consortium (IBDGC) was created in 2002 by the National Institute of Diabetes, Digestive and Kidney diseases (NIDDK) to advance knowledge on the inflammatory bowel diseases, specifically Crohn's Disease and Ulcerative Colitis. The Consortium consists of six genetic research centers (GRC) and a data coordinating center (DCC) that prospectively recruits a combination of cases, controls, and trios to gather a large collection of samples and linked phenotype information. DNA samples are used to conduct genetic linkage and association studies. For more information please see https://ibdgc.org/. Control samples from the following cohorts were included in the Epi25 GWAS: The University of Pittsburgh School of Medicine (PI: Richard Duerr), The Johns Hopkins Hospital (PI: Steven Brant), The Icahn School of Medicine at Mount Sinai (PI: Judy Cho), and Cedars Sinai (PI: Dermot McGovern, Stephan Targan). All samples were genotyped on the GSA-MD v1.0 by the Broad Genomics Platform.

We thank the National Institute of Diabetes and Digestive and Kidney Diseases (NIDDK) IBD Genetics Consortium (IBDGC) supported by The Helmsley Charitable Trust and the Centers for Common Disease Genomics (NHGRI CCDG). Genotyping of samples on the GSA-MD v1 was done by the Broad Genomics Platform and supported by the NHGRI CCDG grant (1UM1HG008895). GSA-MD v1.0 data for these samples is available from dbGaP/AnVIL under study accession number phs001642.

Mass General Brigham (MGB) Biobank

Description/Acknowledgements/Funding:
The MGB (formerly Partners) Biobank (https://biobank.massgeneralbrigham.org/), launched in 2010, is a biorepository of consented patients samples at Mass General Brigham (parent organization of Massachusetts General Hospital and Brigham and Women's Hospital). The Biobank has enrolled >100K individuals to study how genes, lifestyle, and other factors affect people's health and contribute to disease. As part of the NHGRI's Centers for Common Disease Genomics, Broad Institute of MIT and Harvard generated genetic data for ~13,500 individuals from the MGB Biobank. We gratefully acknowledge the participants and leadership team of the MGB Biobank, funding support from the NHGRI CCDG (1UM1HG008895), and generation of new genotype data (Illumina Infinium GSA-MD v1) by the Broad Genomics Platform. GSA-MD v1.0 data for these samples is available from dbGaP under study accession number phs002018.v1.p1.

Classification of epilepsy
We classified seizures and epilepsy syndromes following the International League Against Epilepsy terminology, in accordance with our previously published GWAS.[1] Epilepsy specialists assessed phenotype for all cases at the source centre. Patients with epilepsy were assigned to one of three phenotypic categories: genetic generalised epilepsy, focal epilepsy, or unclassified epilepsy.
Criteria for genetic generalised epilepsy were tonic-clonic, absence, or myoclonic seizures with generalised spike–wave discharges on EEG and no evidence of an acquired cause. In rare instances the criterion for a diagnostic EEG was waived when clear clinical evidence suggested myoclonic or absence seizures with tonic-clonic seizures, and no evidence for an acquired cause. The International League Against Epilepsy has adopted the term genetic generalised epilepsy for syndromes previously known as idiopathic or primary generalised epilepsies, in view of strong evidence for a genetic basis from genetic epidemiological and twin studies and an absence of identified acquired factors.[25]
We included patients with a confirmed diagnosis of focal epilepsy, including cases with focal structural brain lesions. These cases were predominantly adults, and as such, cases of benign epilepsy of childhood with centro-temporal spikes were not specifically included.

Unclassified epilepsy consisted of patients in whom there was neither electroclinical evidence for generalised epilepsy nor evidence for a focal seizure onset. Additionally, cases with evidence for both generalised and focal epilepsy were included in the unclassified category.
Where possible, we used EEG, MRI and clinical history to further refine the following subphenotypes: juvenile myoclonic epilepsy (JME), childhood absence epilepsy (CAE), juvenile absence epilepsy (JAE), generalized tonic-clonic seizures alone (GTCSA), non-lesional focal epilepsy, focal epilepsy with hippocampal sclerosis (HS) and focal epilepsy with lesion other than HS.[25]

Source code for Figure 2

ILAE_SourceData_Fig2_Rscript.R

```
#Rscript for generating Figure 2 for ILAE Consortium GWAS 3.0

#set-up
cor.dir <- " "

library(tidyverse)
library(corrplot)
library(readxl)

# read in corr data
cor.mat <- read_excel(file.path(cor.dir, "ILAE_SourceData_Fig2.xlsx"), sheet=1) %>% t
pval.mat <- read_excel(file.path(cor.dir, "ILAE_SourceData_Fig2.xlsx"), sheet=2) %>% t

names <- c("All\nEpilepsy", "Focal", "GGE")

# check data as matrix
colnames(cor.mat) <- names
as.matrix(cor.mat)

colnames(pval.mat) <- names
as.matrix(pval.mat)

# generate plot
corrplot(as.matrix(t(cor.mat)), p.mat = as.matrix(t(pval.mat)),
        method = "square", type = "full", tl.col = 'black',
        tl.cex = 1.4, tl.srt = 90 ,
        sig.level = c(0.0009, 0.05), pch.cex = 1.8, col = COL2('RdBu', 10),
        insig = 'label_sig', pch.col = 'grey40', is.corr = TRUE)
```

Supplementary References

1. International League Against Epilepsy Consortium on Complex Epilepsies. Genome-wide mega-analysis identifies 16 loci and highlights diverse biological mechanisms in the common epilepsies. Nat Commun 2018;9:5269.
2. Mullins N, Forstner AJ, O'Connell KS, et al. Genome-wide association study of more than 40,000 bipolar disorder cases provides new insights into the underlying biology. Nat Genet 2021;53:817-829.
3. Demontis D, Walters RK, Martin J, et al. Discovery of the first genome-wide significant risk loci for attention deficit/hyperactivity disorder. Nat Genet 2019;51:63-75.
4. Grove J, Ripke S, Als TD, et al. Identification of common genetic risk variants for autism spectrum disorder. Nat Genet 2019;51:431-444.
5. Trubetskoy V, Pardinas AF, Qi T, et al. Mapping genomic loci implicates genes and synaptic biology in schizophrenia. Nature 2022;604:502-508.
6. Howard DM, Adams MJ, Clarke TK, et al. Genome-wide meta-analysis of depression identifies 102 independent variants and highlights the importance of the prefrontal brain regions. Nat Neurosci 2019;22:343-352.
7. Skotte L, Fadista J, Bybjerg-Grauholm J, et al. Genome-wide association study of febrile seizures implicates fever response and neuronal excitability genes. Brain 2022;145:555-568.

8. Nalls MA, Blauwendraat C, Vallerga CL, et al. Identification of novel risk loci, causal insights, and heritable risk for Parkinson's disease: a meta-analysis of genome-wide association studies. Lancet Neurol 2019;18:1091-1102.
9. Wightman DP, Jansen IE, Savage JE, et al. A genome-wide association study with 1,126,563 individuals identifies new risk loci for Alzheimer's disease. Nat Genet 2021;53:1276-1282.
10. Malik R, Chauhan G, Traylor M, et al. Multiancestry genome-wide association study of 520,000 subjects identifies 32 loci associated with stroke and stroke subtypes. Nat Genet 2018;50:524-537.
11. Meng W, Adams MJ, Hebert HL, Deary IJ, McIntosh AM, Smith BH. A Genome-Wide Association Study Finds Genetic Associations with Broadly-Defined Headache in UK Biobank (N=223,773). EBioMedicine 2018;28:180-186.
12. International Multiple Sclerosis Genetics Consortium. Multiple sclerosis genomic map implicates peripheral immune cells and microglia in susceptibility. Science 2019;365.
13. Chiou J, Geusz RJ, Okino ML, et al. Interpreting type 1 diabetes risk with genetics and single-cell epigenomics. Nature 2021;594:398-402.
14. Morris DL, Sheng Y, Zhang Y, et al. Genome-wide association meta-analysis in Chinese and European individuals identifies ten new loci associated with systemic lupus erythematosus. Nat Genet 2016;48:940-946.
15. Savage JE, Jansen PR, Stringer S, et al. Genome-wide association meta-analysis in 269,867 individuals identifies new genetic and functional links to intelligence. Nat Genet 2018;50:912-919.
16. Jansen PR, Watanabe K, Stringer S, et al. Genome-wide analysis of insomnia in 1,331,010 individuals identifies new risk loci and functional pathways. Nat Genet 2019;51:394-403.
17. Karlsson Linner R, Biroli P, Kong E, et al. Genome-wide association analyses of risk tolerance and risky behaviors in over 1 million individuals identify hundreds of loci and shared genetic influences. Nat Genet 2019;51:245-257.
18. Mahajan A, Taliun D, Thurner M, et al. Fine-mapping type 2 diabetes loci to single-variant resolution using high-density imputation and islet-specific epigenome maps. Nat Genet 2018;50:1505-1513.
19. van der Harst P, Verweij N. Identification of 64 Novel Genetic Loci Provides an Expanded View on the Genetic Architecture of Coronary Artery Disease. Circ Res 2018;122:433-443.
20. Frei O, Holland D, Smeland OB, et al. Bivariate causal mixture model quantifies polygenic overlap between complex traits beyond genetic correlation. Nat Commun 2019;10:2417.
21. de Leeuw CA, Mooij JM, Heskes T, Posthuma D. MAGMA: generalized gene-set analysis of GWAS data. PLoS Comput Biol 2015;11:e1004219.
22. Watanabe K, Taskesen E, van Bochoven A, Posthuma D. Functional mapping and annotation of genetic associations with FUMA. Nat Commun 2017;8:1826.
23. Zhu, Zhihong, Futao Zhang, Han Hu, Andrew Bakshi, Matthew R. Robinson, Joseph E. Powell, Grant W. Montgomery, et al. 2016. Integration of Summary Data from GWAS and EQTL Studies Predicts Complex Trait Gene Targets. Nature Genetics 48 (5): 481–87.
24. Freytag S, Burgess R, Oliver KL, Bahlo M. brain-coX: investigating and visualising gene co-expression in seven human brain transcriptomic datasets. Genome Med 2017;9:55.
25. Scheffer IE, Berkovic S, Capovilla G, Connolly MB, French J, Guilhoto L, et al. ILAE classification of the epilepsies: Position paper of the ILAE Commission for Classification and Terminology. Epilepsia 2017; 58:512-521
