# Supplementary material for: GWAS meta-analysis of over 29,000 people with epilepsy identifies 26 risk loci and subtype-specific genetic architecture
Source: Nat Genet. 2023 Aug 31;55(9):1471–82. doi: 10.1038/s41588-023-01485-w (PMC10484785; doi:10.1038/s41588-023-01485-w)
Supplement: Supplementary file 2 — Reporting Summary [file 41588_2023_1485_MOESM2_ESM.pdf]

nature portfolio

Corresponding author(s): Berkovic SF, Cavalleri GL, Koeleman BPC

Last updated by author(s): 6/19/2023

Reporting Summary

Nature Portfolio wishes to improve the reproducibility of the work that we publish. This form provides structure for consistency and transparency in reporting. For further information on Nature Portfolio policies, see our Editorial Policies and the Editorial Policy Checklist.

Statistics

For all statistical analyses, confirm that the following items are present in the figure legend, table legend, main text, or Methods section.

n/a	Confirmed	
☐	☒	The exact sample size (*n*) for each experimental group/condition, given as a discrete number and unit of measurement
☒	☐	A statement on whether measurements were taken from distinct samples or whether the same sample was measured repeatedly
☐	☒	The statistical test(s) used AND whether they are one- or two-sided<br>*Only common tests should be described solely by name; describe more complex techniques in the Methods section.*
☐	☒	A description of all covariates tested
☐	☒	A description of any assumptions or corrections, such as tests of normality and adjustment for multiple comparisons
☐	☒	A full description of the statistical parameters including central tendency (e.g. means) or other basic estimates (e.g. regression coefficient) AND variation (e.g. standard deviation) or associated estimates of uncertainty (e.g. confidence intervals)
☐	☒	For null hypothesis testing, the test statistic (e.g. *F*, *t*, *r*) with confidence intervals, effect sizes, degrees of freedom and *P* value noted<br>*Give P values as exact values whenever suitable.*
☒	☐	For Bayesian analysis, information on the choice of priors and Markov chain Monte Carlo settings
☒	☐	For hierarchical and complex designs, identification of the appropriate level for tests and full reporting of outcomes
☐	☒	Estimates of effect sizes (e.g. Cohen's *d*, Pearson's *r*), indicating how they were calculated

Our web collection on statistics for biologists contains articles on many of the points above.

Software and code

Policy information about availability of computer code

Data collection	Genotyping was performed at various sites as detailed in the Methods and Supplementary Table 1. Genotypes were harmonized to the Haplotype Reference Consortium (HRC) panel v1.1 using tools available from https://www.well.ox.ac.uk/~wrayner/tools/ prior to imputation using one of the two following imputation servers:<br>1. Wellcome Sanger Institutes imputation server: https://imputation.sanger.ac.uk<br>2. Michigan Imputation server: https://imputationserver.sph.umich.edu/
Data analysis	No custom code was used in this study. We used the following public and freely available software tools to preform the reported analyses:<br>Eagle v2.4.1 for haplotype phasing;<br>PBWT v3.1 for imputation;<br>Plink v1.9 for genotype data manipulation/QC;<br>Saige v0.38 for generalized mixed models;<br>METAL v2020-05-05 for meta-analyses;<br>Metasoft v2.0.0 for generating m-values for PM-Plots;<br>R v3.6.0 for generating Manhattan and correlation plots;<br>ASSET v2.2.0 for pleiotropy detection;<br>CookHLA v1.0.1 for HLA imputation;<br>HLA Analysis Toolkit (HATK) v1.2 for HLA association analysis<br>ANNOVAR v2017-07-17 for SNP annotation;<br>MTAG v1.0.8 for multi-trait analysis;<br>FUMA v1.3.8 for gene mapping;<br>MAGMA v1.08 for gene set analyses;

FUSION v3 for TWAS;
GWAMA v2.2.2 for sex-specific GWAS
LDAK v5.2 for SNP-based heritability analyses;
MiXeR v1.2.0 for causal mixture models;
LDSC v.1.01 for cross-train genetic correlations;
Relevant references are listed throughout the manuscript for all above stated tools and software.

For manuscripts utilizing custom algorithms or software that are central to the research but not yet described in published literature, software must be made available to editors and reviewers. We strongly encourage code deposition in a community repository (e.g. GitHub). See the Nature Portfolio guidelines for submitting code & software for further information.

Data

Policy information about availability of data

All manuscripts must include a data availability statement. This statement should provide the following information, where applicable:

- Accession codes, unique identifiers, or web links for publicly available datasets
- A description of any restrictions on data availability
- For clinical datasets or third party data, please ensure that the statement adheres to our policy

The GWAS summary statistics data that support the findings of this study (for both multi-ancestry and European-only analyses) are publicly available at https://www.epigad.org/ and in the NHGRI-EBI GWAS Catalog at https://www.ebi.ac.uk/gwas/ (accession IDs: GCST90271608, GCST90271609, GCST90271610, GCST90271611 ,GCST90271612, GCST90271613, GCST90271614, GCST90271615, GCST90271616, GCST90271617, GCST90271618, GCST90271619 & GCST90271620). Individual-level GSA-MD v1.0 data for the Epi25 case samples and HKOS control samples are available in dbGaP/AnVIL under phs001489.v2.p2. GSA-MD v1.0 data for Genomic Psychiatry Cohort (GPC) control samples data will be made available in dbGAP/AnVIL under study phs002041. Individual-level SNP genotype data for other cohorts used as controls in the Epi25 analyses are accessible via an application through the THL Biobank portal (https://thl-biobank.elixir-finland.org/) for FINRISK, and in dbGaP/AnVIL under study accession numbers phs001642 (NIDDK IBDGC) and phs002018.v1.p1 (MGB Biobank) (see Supplementary information for more details). Data relating to UKBiobank is available via application to UKBiobank (https://www.ukbiobank.ac.uk/enable-your-research/apply-for-access). The FinnGen data can be accessed through the Fingenious services (https://site.fingenious.fi/en/) managed by FINBB: release R6. The summary statistics of the Japanese GWAS in this study are publicly available from the National Bioscience Database Center (https://biosciencedbc.jp/en) under research ID: hum0014. We also accessed data from the following online database: www.DGidb.com (accessed on 26-11-2021).

Human research participants

Policy information about studies involving human research participants and Sex and Gender in Research.

Reporting on sex and gender	Self-identified gender was collected upon recruitment of both cases and controls. Biological sex was subsequently determined genetically and used for downstream analyses.
Population characteristics	Diagnoses: 29944 patients with epilepsy, 52538 controls<br>Genotypic ancestry (cases & controls): 69995 European, 5306 Asian, 7181 African<br>Sample age data was not available.
Recruitment	Case and control samples were recruited from tertiary hospital and academic research centres. All cases were diagnosed with epilepsy syndrome according to the same international guidelines and classification system, however, it is possible that the application of diagnostic criteria across cohorts may slightly differ. This ascertainment bias may have resulted in a reduction to the overall power of the study and the generalizability of results.
Ethics oversight	All contributing case and control sites collected samples following local IRB/ethics committee approval. A full list of approval bodies can be found in Supplementary Table 1.

Note that full information on the approval of the study protocol must also be provided in the manuscript.

Field-specific reporting

Please select the one below that is the best fit for your research. If you are not sure, read the appropriate sections before making your selection.

☒ Life sciences ☐ Behavioural & social sciences ☐ Ecological, evolutionary & environmental sciences

For a reference copy of the document with all sections, see nature.com/documents/nr-reporting-summary-flat.pdf

Life sciences study design

All studies must disclose on these points even when the disclosure is negative.

Sample size	Sample size was not predetermined, however, we note that this study is almost twice the size of the previous largest epilepsy GWAS published in 2018.
Data exclusions	We excluded poorly genotyped SNPs and outlier samples according to the various QC parameters which are described in our methods.

Replication	As this is the largest study of common variants in epilepsy to date we did not have additional samples to replicate the significant variants in this study. However we have demonstrated reproducibility of our previous study findings and expect to reproduce this study findings with the addition of new samples in future work.
Randomization	There was no randomization in this study. Cases were grouped according to electroclinical phenotypes in epilepsy while controls were unscreened population samples.
Blinding	Samples were coded at collection and phenotypes were collected by separate individuals from analysts, preventing analysts from making genotype-phenotype identification of a study participant.

Reporting for specific materials, systems and methods

We require information from authors about some types of materials, experimental systems and methods used in many studies. Here, indicate whether each material, system or method listed is relevant to your study. If you are not sure if a list item applies to your research, read the appropriate section before selecting a response.

Materials & experimental systems

n/a	Involved in the study	
☒	☐	Antibodies
☒	☐	Eukaryotic cell lines
☒	☐	Palaeontology and archaeology
☒	☐	Animals and other organisms
☒	☐	Clinical data
☒	☐	Dual use research of concern

Methods

n/a	Involved in the study	
☒	☐	ChIP-seq
☒	☐	Flow cytometry
☒	☐	MRI-based neuroimaging
